# Floridean Starch and Floridoside Metabolic Pathways of *Neoporphyra haitanensis* and Their Regulatory Mechanism under Continuous Darkness

**DOI:** 10.3390/md19120664

**Published:** 2021-11-26

**Authors:** Yahui Yu, Xuli Jia, Wenlei Wang, Yuemei Jin, Weizhi Liu, Dongmei Wang, Yunxiang Mao, Chaotian Xie, Tao Liu

**Affiliations:** 1 College of Marine Life Sciences, Ocean University of China, Qingdao 266003, China; yyhsdhz3390@163.com (Y.Y.); 18734539035@163.com (X.J.); mgbl_sr@ouc.edu.cn (Y.J.); liuweizhi@ouc.edu.cn (W.L.); wangdm@ouc.edu.cn (D.W.); 2 Fisheries College, Jimei University, Xiamen 361021, China; wlwang@jmu.edu.cn; 3 Fujian Engineering Research Center of Aquatic Breeding and Healthy Aquaculture, Xiamen 361021, China; 4 Key Laboratory of Marine Genetics and Breeding (Ocean University of China), Ministry of Education, Qingdao 266003, China; 5 Key Laboratory of Utilization and Conservation for Tropical Marine Bioresources (Hainan Tropical Ocean University), Ministry of Education, Sanya 572022, China; 6 Yazhou Bay Innovation Institute, Hainan Tropical Ocean University, Sanya 572024, China; 7 Laboratory for Marine Biology and Biotechnology, Qingdao Pilot National Laboratory for Marine Science and Technology, Qingdao 266237, China; 8 College of Ocean and Earth Sciences, Xiamen University, Xiamen 361102, China; 9 Southern Marine Science and Engineering Guangdong Laboratory (Zhuhai), Zhuhai 519000, China

**Keywords:** darkness, floridean starch, floridoside, metabolic pathway, *Neoporphyra haitanensis*

## Abstract

Floridean starch and floridoside are the main storage carbohydrates of red algae. However, their complete metabolic pathways and the origin, function, and regulatory mechanism of their pathway genes have not been fully elucidated. In this study, we identified their metabolic pathway genes and analyzed the changes in related gene expression and metabolite content in *Neoporphyra haitanensis* under continuous dark conditions. Our results showed that genes from different sources, including eukaryotic hosts, cyanobacteria, and bacteria, were combined to construct floridean starch and floridoside metabolic pathways in *N. haitanensis*. Moreover, compared with those in the control, under continuous dark conditions, floridean starch biosynthesis genes and some degradation genes were significantly upregulated with no significant change in floridean starch content, whereas floridoside degradation genes were significantly upregulated with a significant decrease in floridoside content. This implies that floridean starch content is maintained but floridoside is consumed in *N. haitanensis* under dark conditions. This study elucidates the “floridean starch–floridoside” metabolic network and its gene origins in *N. haitanensis* for the first time.

## 1. Introduction

Land plants and photosynthetic algae convert light energy into chemical energy through photosynthesis and store it as organic compounds, such as carbohydrates and amino acids [1,2]. Different species of algae use different storage carbohydrates. The main storage carbohydrates in green algae and land plants are starch and sucrose [3,4], in red algae are floridean starch and floridoside, and in brown algae are mannitol and laminaran [5]. As primary energy sources, these storage carbohydrates play an important role in the life cycle of land plants and algae.

In the leaves of green plants, starch accumulates during the day and degrades at night to provide carbon and energy for plant development [6]. Specifically, maltose produced by starch degradation is converted to hexose phosphates, which serve as substrates for sucrose synthesis [7]. Meanwhile, in red algae, *Porphyridium purpureum* (Rhodophyta) retains a considerable amount of floridean starch after a few weeks of dark incubation [8], whereas the floridean starch content of *Gracilariopsis lemaneiformis* (Rhodophyta) decreases considerably in the dark [9]. Hence, different species of red algae have different starch metabolism mechanisms in a dark environment. Moreover, during a 24 h dark period, floridoside content reportedly decreases slowly in *Porphyra purpurea* (Rhodophyta) [10]. Floridoside is a dynamic carbon source that serves as a carbon precursor for starch and cell wall polysaccharide biosynthesis in *Porphyridium* sp. [11]. Based on these results, we speculated that certain species of red algae can accumulate floridean starch under dark conditions, while the synthetic precursor might originate from floridoside.

Floridian starch is a semi-crystalline polysaccharide similar to starch and differs between land plants and green algae based on amylopectin and amylose proportions [3]. In addition to serving as photosynthetic products for red algae, floridean starch may also facilitate the efficient production of hydroxymethylfurfural (HMF) [12]. Floridean starch metabolism genes have been annotated in the genomes of red algae, such as *P. purpureum*, *Chondrus crispus*, *Porphyra umbilicalis*, and *Gracilaria changii* (Rhodophyta) [13,14,15,16]. However, the complete floridean starch metabolic pathway, as well as the regulatory mechanisms under dark conditions in *Neoporphyra haitanensis* (formerly *Pyropia haitanensis*) (Rhodophyta), remain unknown.

Floridoside (α-d-galactopyranosyl-(1,2)-glycerol), a natural galactosyl glycerol, is the main common soluble photosynthetic molecule synthesized in red algae cytoplasm, and exhibits prebiotic characteristics in vitro [17,18]. Isofloridoside, an enantiomeric form of floridoside, has two isomeric forms: D- and L-isofloridoside [19]. (Iso)floridoside metabolic pathways have been characterized by crude and purified enzyme preparations in various red algae, including *Neoporphyra perforata* (formerly *Pyropia perforata*) (Rhodophyta), *Poterioochromonas malhamensis* (Ochrophyta, Chrysophyceae), *Gracilaria tenuistipitata* (Rhodophyta), and *Galdieria sulphuraria* (Rhodophyta) [20]. Additionally, the trehalose phosphate synthase (TPS) genes encoding the enzymatic activity of floridoside and isofloridoside phosphate synthase were successively verified in *G. sulphuraria*, *N. haitanensis*, and *P. umbilicalis* [20,21,22]. However, the complete metabolic pathway of floridoside in *N. haitanensis* has not been fully elucidated, nor have the regulatory mechanisms under dark conditions.

*N. haitanensis* (Bangiales, Rhodophyta), a species native to China and widely distributed on its southeastern coast, is commonly consumed as a food source in Asian countries and has the highest annual production among all “nori” species [23]. Its economic value and unique physiological characteristics have attracted great interest in the study of this species, which is considered a genetic and physiological model for analyzing the stress resistance of intertidal red seaweed [24]. Such studies have shown that desiccated gametophytes of *N. haitanensis*, placed in the freezer (−20 °C) for 30 days in dark conditions, can survive after rehydration [25]. Additionally, 3-day dark treatment reportedly enhances spore release and increases the sensitivity of *Neopyropia yezoensis* (formerly *Pyropia yezoensis*) gametophytes to wounding [26]. Meanwhile, limited information is available regarding the metabolic pathways of floridean starch and floridoside, which provide energy for the growth and development of *N. haitanensis* as storage carbohydrates. 

In the present study, using bioinformatics analysis, the floridean starch and floridoside metabolic pathways in *N. haitanensis* were constructed, and the origins of the involved genes were proposed. Through comparative metabolite and transcriptomic analyses under light and dark conditions, the gene regulatory mechanisms in these pathways, as well as the characteristics of metabolite changes, were revealed. Hence, this study enhances our understanding of floridean starch and floridoside metabolism in *N. haitanensis* and provides valuable information for exploring the utilization of algal carbohydrates in contexts such as ecological adaptation and breeding. 

## 2. Results

### 2.1. Identification of Candidate Genes

The genes (or families) involved in the floridean starch and floridoside metabolic pathways of eight red algae species (*Chondrus crispus*, *Cyanidioschyzon merolae*, *Gracilariopsis chorda*, *Galdieria sulphuraria*, *Porphyridium purpureum*, *Neopyropia yezoensis*, *Neoporphyra haitanensis*, and *Porphyra umbilicalis*) were identified (Table S1). Three shared upstream genes, glucose-6-phosphate isomerase (*GPI*), phosphoglucomutase (*PGM*), and UDP-glucose pyrophosphorylase (*UGP*), were involved in the conversion of fructose-6-phosphate to UDP-glucose. Subsequently, UDP-glucose enters the floridean starch and floridoside metabolic pathways: the floridean starch metabolic pathway involves starch synthase (*SS_UDPG_*), branching enzyme (*BE*), isoamylase (*ISA*), phosphoglucan water dikinase (*PWD*), glucan water dikinase (*GWD*), dual-specificity phosphatase starch excess 4 (SEX4/laforin), β-amylase (*BAM*), pullulanase (*PUL*), α-amylase (*AMY*), transglucosidase (*DPE2*), and phosphorylase (*PHO*); and the floridoside metabolic pathway route involves UDP-glucose-4-epimerase (*GALE*), glycerol kinase (*GK*), glycerol-3-phosphate dehydrogenase (*GPDH*), trehalose-6-phosphate synthase (*TPS*), trehalose-6-phosphate phosphatase (*TPP*), α-galactosidase (*GLA*), and galactose-1-phosphate uridylyltransferase (*GALT*).

**Figure 1:**
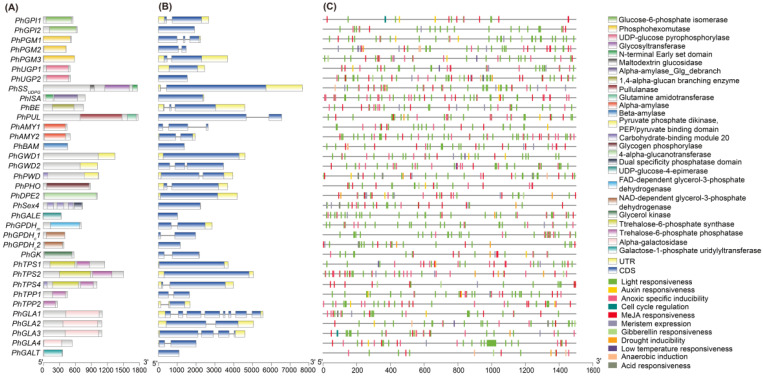
The domain arrangements, gene structures, and cis-acting elements of floridean starch and floridoside metabolic pathway genes identified in *Neoporphyra haitanensis*. (**A**) Domain arrangements. The different colored blocks represent the different types of conserved domains. Amino acid length scale is shown at the bottom. (**B**) Exon–intron gene structures. The boxes filled with blue represent exons; solid black lines represent introns. The untranslated regions (UTRs) are indicated by yellow boxes. Nucleotide length scale is shown at the bottom. (**C**) Analysis of 1.5 kb upstream cis-acting gene elements. The different colored blocks represent the different types of cis-acting elements. Nucleotide length scale is shown at the bottom.

In total, 35 genes involved in floridean starch and floridoside metabolic pathways of *N. haitanensis* were identified (Table 1 and Table S2). These genes were members of 21 gene families, and 12 genes were identified as single gene copies (Table 1). The physicochemical properties and sequence feature of these genes were analyzed by the ProtParam tool and TBtools (Table 1). The conserved domains were verified using the CDD program and are shown in Figure 1A. Among these genes, 13 were intronless, 16 had one intron, and only six genes had two or more introns (Table 1, Figure 1B). Four categories of cis-elements were identified (Figure 1C), including light control elements (light responsiveness), phytohormone responsive elements (auxin, gibberellin, abscisic acid, and methyl jasmonate (MeJA) responsiveness), abiotic and biotic stress-responsive elements (drought inducibility, low temperature responsiveness, anoxic specific inducibility, and anaerobic induction), and plant development-related cis-elements (cell cycle regulation and meristem expression). Among these four categories, the proportion of the light control element category was the highest.

**Figure 2:**
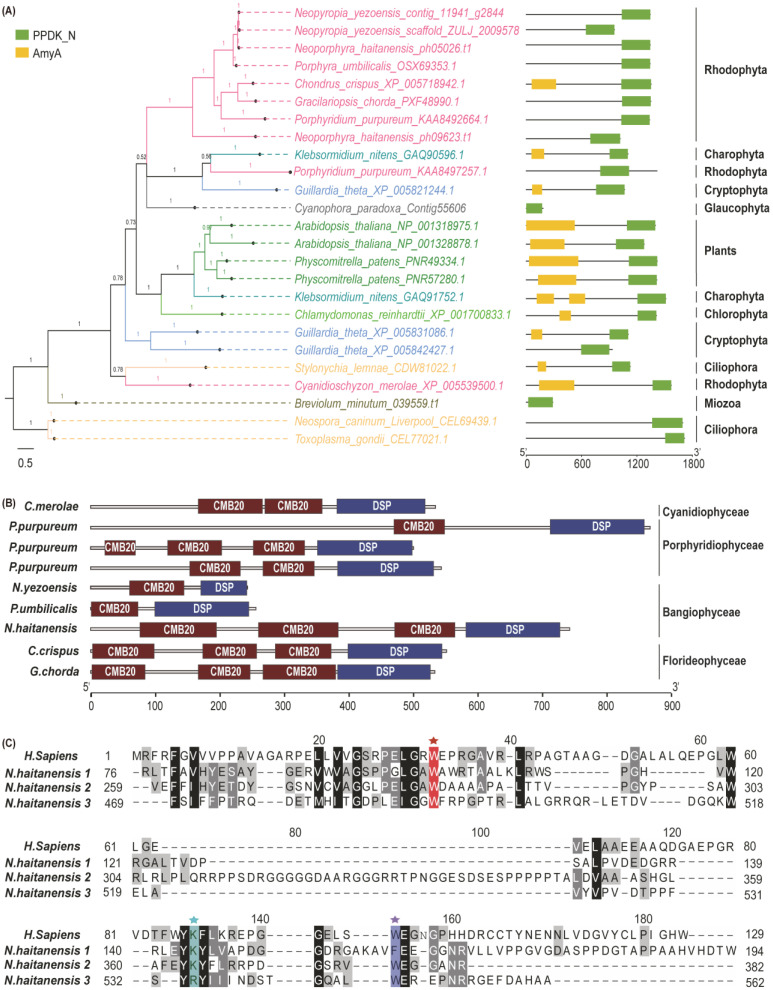
Domain arrangement and sequence alignment of GWD and SEX4. (**A**) Bayesian phylogenetic tree and domain arrangement of GWD amino acid sequences. All protein accession numbers are listed in Table S3. The domain architecture of proteins is shown, and different colored blocks represent different types of conserved domains. Amino acid length scale is shown at the bottom. PPDK_N, pyruvate phosphate dikinase; AmyA, α-amylase. (**B**) Domain arrangement of SEX4 of seven red algae species. CBM20, carbohydrate-binding module 20; DSP, dual-specificity phosphatase domain. (**C**) Amino acid sequence alignment of CMB20 sequences from SEX4 of *Neoporphyra haitanensis* and laforin of *Homo sapiens*. The CBM20 positions involved in starch-binding are indicated by stars (Trp32, red; Lys87, blue; Trp99, purple).

The distribution of starch-binding domains in the genes involved in floridean starch and floridoside metabolic pathways was further analyzed. Figure 1A shows PhPWD contained a carbohydrate-binding module 20 (CBM20) domain and a PEP/pyruvate binding domain of pyruvate phosphate dikinase (PPDK_N). However, PhGWD1 and PhGWD2 contained only a PPDK_N domain at the N terminal. Then, we compared the domain arrangement of GWD in *N. haitanensis* with that in other algae and land plants and found that the N terminal of GWD in land plants, chlorophytes, cryptophytes, and charophytes contained an α-amylase domain (Figure 2A). However, the α-amylase domain was missing in dinoflagellates, glaucophytes, ciliates, and most red algae (Figure 2A). There were three duplicated carbohydrate-binding module 20 (CBM20) domains in the N terminal of PhSEX4 (Figure 2B). The phenomenon of over one CBM20 domain in the SEX4 is also present in *C. merolae*, *P. purpureum*, *C. crispus*, and *G. chorda* (Figure 2B). We performed multiple-sequence alignments of the three CBM20 sequences in PHSEX4 and found that Trp99 and Lys87 (*Homo sapiens laforin* numbering) [27], the residues identified as responsible for binding *α*-glucans, lacked the first and second CBM20 domains of PhSEX4 (Figure 2C). In addition, only the N terminal of PhTPS4 contained a CBM20 domain in PhTPS family proteins (Figure 1A).

### 2.2. The Origin of Genes Involved in Floridean Starch and Floridoside Metabolic Pathways of N. haitanensis

**Figure 3:**
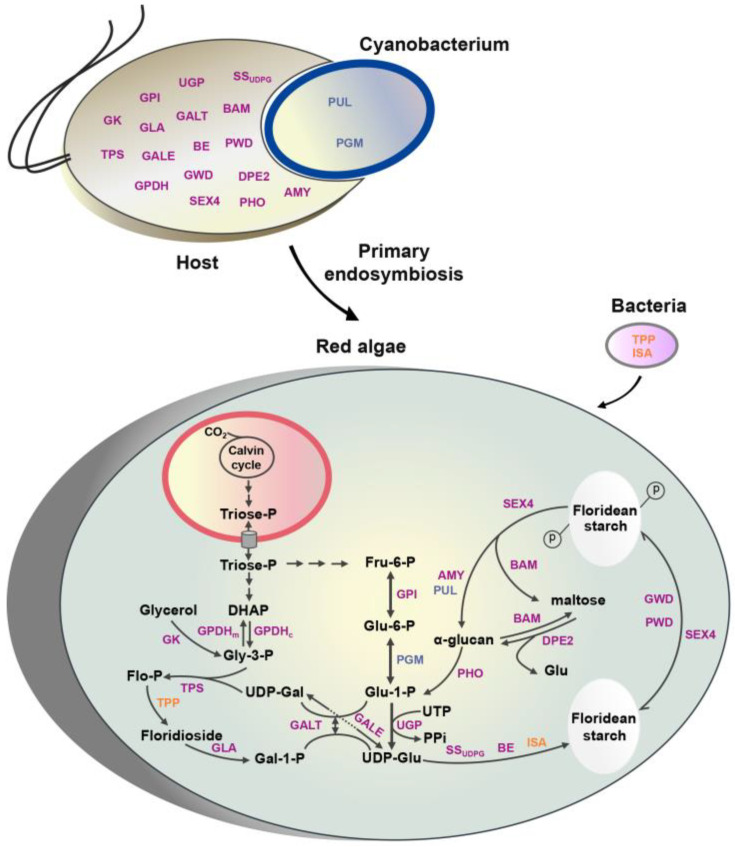
Floridean starch and floridoside metabolic pathways. Our model was constructed based on previously published concepts [13,28,29]. The phylogenetic origin of each enzyme is indicated as follows: blue color = cyanobacterial origin, purple color = eukaryotic host origin, yellow color = bacterial origin. In red algae plastids, CO_2_ is assimilated into triose-phosphates (TPs), most of which are transported to the cytoplasm. These TPs are converted to fructose-6-phosphate (Fru-6-P) and dihydroxyacetone phosphate (DHAP) to sustain floridean starch and floridoside synthesis (41). Fru-6-P is transformed in glucose-6-phosphate (Glu-6-P) by glucose-6-phosphate isomerase (GPI) and then converted to glucose-1-phosphate (Glu-1-P) by phosphoglucomutase (PGM). Glu-1-P reacts with UTP, and this reaction is catalyzed by UDPG pyrophosphorylase (UGP), to generate UDP-glucose (UDP-Glu). Then, UDP-glucose can continue in two directions, floridean starch and floridoside biosynthesis. In floridean starch biosynthesis, glucose from UDP-Glu is catalyzed by starch synthase (SS_UDPG_), branching enzyme (BE), and isoamylase (ISA) to generate floridean starch. Starch phosphorylation is the first step of starch degradation, and it is initiated by glucan water dikinase (GWD) and phosphoglucan water dikinase (PWD) at glucose C6 and C3 positions, respectively. Dual-specificity phosphatase starch excess 4 (SEX4) dephosphorylates starch granule surfaces and soluble phosphoglucans, which enables the glucan chains to be further degraded by β-amylase (BAM). Phosphorylated starch is then attacked by BAM, pullulanase (PUL), and α-amylase (AMY), resulting in the generation of maltose and α-glucan. Glucose is released and transferred from maltose by transglucosidase (DPE2). α-glucan can also be attacked by BAM and phosphorylase (PHO), which releases maltose and Glu-1-P, respectively. Floridoside synthesis requires two substrates, UDP-galactose (UDP-Gal) and glycerol-3-phosphate (Gly-3-P). UDP-Gal is isomerized by UDP-Glu under the catalysis of UDP-glucose-4-epimerase (GALE). Gly-3-P is derived by the glycerol kinase (GK)- mediated phosphorylation of glycerol. It can also be derived by the NAD-dependent glycerol-3-phosphate dehydrogenase (GPDH_C_)-mediated reduction of dihydroxyacetone phosphate (DHAP). Gly-3-p can also be converted into DHAP under the catalysis of FAD-dependent glycerol-3-phosphate dehydrogenase (GPDH_m_). With UDP-Glu and Gly-3-P as substrates, floridoside is synthesized by trehalose-6-phosphate synthase (TPS) and trehalose-6-phosphate phosphatase (TPP). During floridoside degradation, floridoside is degraded to galactose-1-P (Gal-1-P) by α-galactosidase (GLA), after which, Gal-1-P and UDP-Glu are reversibly converted into Glu-1-P and UDP-Gal by galactose-1-phosphate uridylyltransferase (GALT).

The full-length amino acid sequences of genes involved in the floridean starch and floridoside metabolic pathways from archaea, bacteria, cyanobacteria, fungi, oomycetes, protozoa, tracheophytes, and eukaryotic algae were used to construct phylogenetic trees by using Bayesian methods. The phylogenetic trees indicated that the upstream genes *PhGPIs*, *PhUGPs*, and *PhPGMs*, shared by the two metabolic pathways, were widely distributed among diverse algae species. Among them, *PhGPIs* and *PhUGPs* may have been derived from primary endosymbiotic eukaryotic hosts, and *PhPGMs* may have originated from primary endosymbiotic cyanobacteria (Figure 3 and Figures S1–S3). Among the genes involved in floridean starch metabolism, *PhSS_UDPG_*, *PhBE*, *PhGWDs*, *PhPWD*, *PhSEX4*, *PhAMYs*, *PhBAM*, *PhPHO*, and *PhDPE2* may have been derived from primary endosymbiotic eukaryotic hosts, *PhPUL* may have originated from primary endosymbiotic cyanobacteria, whereas *PhISA* may have originated from bacteria via horizontal gene transfer (Figure 3 and Figures S4–S13). Among the genes related to the floridoside metabolic pathway, *PhGALE, PhGK, PhGPDHs, PhTPSs, PhGLAs*, and *PhGALT* may have been derived from primary endosymbiotic eukaryotic hosts, whereas *PhTPPs* may have originated from bacteria via horizontal gene transfer (Figure 3 and Figures S14–S19). Detailed information on all gene evolutionary trees is shown in Figures S1–S19, and the sequence information is shown in Table S3.

### 2.3. Photochemical Reactions under Dark Conditions

**Figure 4:**
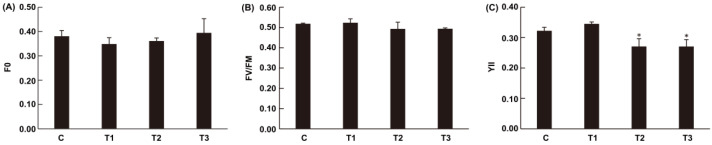
Chlorophyll fluorescence parameters under dark conditions. (**A**) Minimal fluorescence (F_0_). (**B**) Maximal PSII quantum yield (F_v_/F_m_). (**C**) Quantum yield of photochemical energy conversion in PSII (YII). Abbreviations: C, control; T1, 6 h after darkness; T2, 24 h after darkness; T3, 48 h after darkness. One-way analysis of variance (ANOVA) and Tukey’s test were performed. Asterisks indicate significant differences between darkness and control for each group (* *p <* 0.05).

The effects of darkness on the photochemical reactions of *N. haitanensis* are shown in Figure 4. No changes were observed in F_0_ or F_v_/F_m_. The thalli reduced by 15.99% (*p <* 0.05) at 24 h and 16.02% (*p <* 0.05) at 48 h in the YII under dark conditions compared to those in the control.

### 2.4. Changes in Metabolite Contents under Dark Conditions

**Figure 5:**
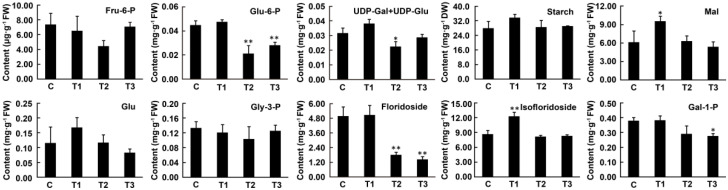
Metabolite contents in floridean starch and floridoside metabolic pathways under the darkness treatment (three replicates per treatment). Error bars represent the standard deviation (SD) for triplicate measurements. One-way analysis of variance (ANOVA) and Tukey’s test were performed. Asterisks indicate significant differences between darkness and control for each group (* *p <* 0.05; ** *p <* 0.01). Abbreviations: C, control; T1, 6 h after darkness; T2, 24 h after darkness; T3, 48 h after darkness; FW, fresh weight; DW, dry weight; Fru-6-P, fructose-6-phosphate; Gly-3-P, glycerol-3-phosphate; Glu, glucose; Glu-6-P, glucose-6-phosphate; Gal-1-P, galactose-1-phosphate; Mal, maltose; UDP-Gal, UDP-galactose; UDP-Glu, UDP-glucose.

The effects of darkness on the metabolite contents of floridean starch and floridoside metabolism are shown in Figure 5. Among the biomass precursors shared by the two pathways, the content of Fru-6-P did not change significantly under dark treatment. The Glu-6-P content decreased by 41.53% (*p <* 0.01) at 24 h and 23.24% (*p <* 0.01) at 48 h of the darkness treatment compared to that in the control. As UDP-Glu and UDP-Gal are isomers, we determined the total content of both and found that their content decreased by 28.54% (*p <* 0.05) compared to that in the control at 24 h and returned to control levels at 48 h of the darkness treatment. Compared to the control, no significant changes in the content of glucose, which is the degradation product of floridean starch, and floridean starch were observed after 6, 24, or 48 h of darkness treatment. The content of maltose, another starch degradation product, transiently increased by 55.36% (*p <* 0.05) at 6 h of the darkness treatment, compared to that in the control, before declining to control levels. The content of Gly-3-P, a precursor involved in the synthesis of (iso)floridoside, did not change significantly under dark treatment. Compared to the control, the floridoside content did not change significantly after 6 h of darkness, but showed a sharp decline under prolonged darkness, with a decrease of 63.20% (*p <* 0.01) at 24 h and 70.39% (*p <* 0.01) at 48 h. At 6 h of darkness, the content of isofloridoside transiently increased by 42.52% (*p <* 0.01) compared to that in the control, after which it declined to control levels. Under dark conditions, the content of Gal-1-P, the degradation product of (iso)floridoside, showed no difference at 6 h and decreased by 23.29% at 24 h and 27.15% (*p <* 0.05) at 48 h compared to that in the control.

**Figure 6:**
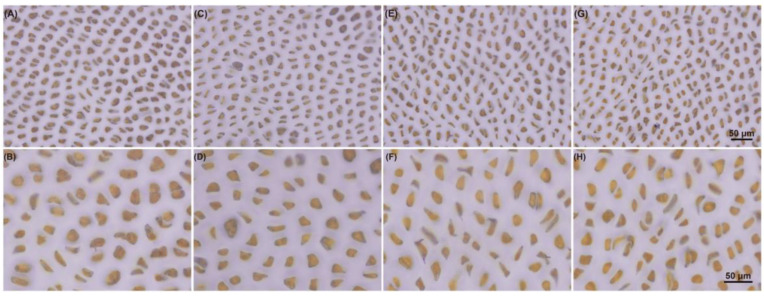
Staining of starch granules. (**A**,**B**), control; (**C**,**D**), 6 h after darkness; (**E**,**F**), 24 h after darkness; (**G**,**H**), 48 h after darkness; scale bar = 50 µm.

Starch iodine staining was also performed to better study the changes in floridean starch under dark conditions. Figure 6 shows that the color and size of starch granules in thalli did not change significantly as the darkness treatment time increased.

### 2.5. Transcriptomic Analysis

To elucidate the metabolic pathways of floridean starch and floridoside, 12 cDNA libraries were constructed from four samples under light (control: 6 h) and dark (6 h, 24 h, and 48 h) treatments. The libraries yielded approximately 110.26 million raw reads (Table S4). All raw data generated in this study were stored in NCBI with the SRA accession number PRJNA670264. After removing the adaptors and unknown or low-quality reads, approximately 99.35 million clean reads were obtained (Table S4). The Q30 of all libraries was higher than 94%, and the GC content of samples from all treatments was ca. 64% (Table S4). Approximately 92.86% of the clean reads could be mapped to the reference genome (Table S5). Gene coverage analysis showed no significant peaks, indicating that no significant publication bias was observed in the sequencing results (Figure S20). The density profile of FPKM was a non-standard normal distribution, and the region area was 1, indicating that the probability sum was approximately 1 (Figure S21). The high correlation between replicates (Pearson correlations > 0.86; Figure S21) indicated that the high-throughput sequencing data can be used for subsequent analyses.

### 2.6. Identification of Differentially Expressed Genes (DEGs) under Dark Conditions

**Figure 7:**
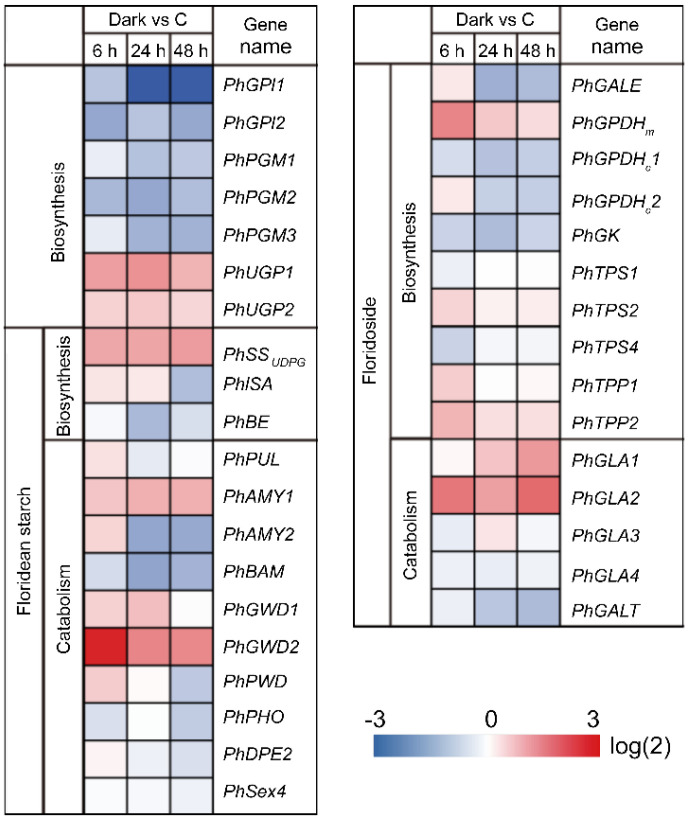
Changes in the expression of genes related to floridean starch and floridoside metabolic pathways under dark conditions. The heat map shows the relative gene expression levels. “Dark vs C” values indicate the ratio of gene expression in darkness and control conditions.

Following the 6 h, 24 h, and 48 h darkness treatments, 2795 (1429 upregulated and 1366 downregulated), 3250 (1599 upregulated and 1651 downregulated), and 2811 (1155 upregulated and 1656 downregulated) DEGs, respectively, were identified in *N. haitanensis* (Figure S22). Functional classification of DEGs was performed using gene ontology (GO) analysis, which divided these DEGs into three categories: biological processes, molecular functions, and cellular components (Figures S23–S25; Table S6). To understand their functions, GO functional enrichment and KEGG pathway enrichment analyses were conducted (Figures S26–S30, Tables S7–S12). Higher GO terms were “polysomal ribosome”, “lipid binding”, and “polysome” in the 24 h dark treatment (Figure S27, Table S8). In the 48 h dark treatment, higher GO terms were “chloroplast part”, “proteasome core complex”, and “plastid part” (Figure S28, Table S9). KEGG enrichment analysis showed that “ko03010: Ribosome” was significantly enriched by DEGs in the 24 h dark treatment (Figure S30, Table S11). As this study focused on the regulatory mechanism of floridean starch and floridoside metabolic pathways in *N. haitanensis* under continuous darkness, changes in the expression of genes associated with floridean starch and floridoside pathways were analyzed and plotted in heat maps (Figure 7 and Table S13).

As shown in Figure 7, among the genes shared by the two pathways, one gene (*PhUGP1*) was markedly upregulated (2.51-fold) and three genes (*PhGPI1*, *PhGPI2*, and *PhPGM2*) were markedly downregulated (2.01-fold, 2.89-fold, and 2.36-fold, respectively) at 6 h of darkness compared to those in the control. At 24 h of darkness, one gene (*PhUGP1*) was markedly upregulated (2.79-fold) and four genes (*PhGPI1*, *PhGPI2*, *PhPGM2*, and *PhPGM3*) were markedly downregulated (7.48-fold, 2.03-fold, 2.90-fold, and 2.54-fold, respectively) compared to those in the control. At 48 h of darkness, one gene (*PhUGP1*) was significantly upregulated (2.05-fold) and four genes (*PhGPI1*, *PhGPI2*, *PhPGM2*, and *PhPGM3*) were markedly downregulated (7.25-fold, 2.87-fold, 2.17-fold, and 2.52-fold, respectively) compared to those in the control.

Among the floridean starch metabolic pathway genes, two genes (*PhSS_UDPG_* and *PhGWD2*) were markedly upregulated (2.29-fold and 7.81-fold, respectively) at 6 h of darkness compared to those in the control. At 24 h of darkness, three genes (*PhSS_UDPG_*, *PhAMY1*, and *PhGWD2*) were markedly upregulated (2.35-fold, 2.14-fold, and 3.14-fold, respectively) and three genes (*PhBE*, *PhAMY2*, and *PhBAM*) were markedly downregulated (2.31-fold, 2.94-fold, and 3.01-fold, respectively) compared to those in the control. At 48 h of darkness, three genes (*PhSS_UDPG_*, *PhAMY1*, and *PhGWD2*) were markedly upregulated (2.54-fold, 2.12-fold, and 3.03-fold, respectively) and three genes (*PhISA*, *PhAMY2*, and *PhBAM*) were markedly downregulated (2.17-fold, 2.83-fold, and 2.48-fold, respectively) compared to those in the control.

Among the floridoside metabolic pathway genes, three genes (*PhGPDH1*, *PhTPP2*, and *PhGLA2*) were markedly upregulated (3.15-fold, 2.03-fold, and 3.59-fold, respectively) at 6 h of darkness compared to those in the control. At 24 h of darkness, one gene (*PhGLA2*) was markedly upregulated (2.46-fold) and three genes (*PhGPDH1*, *PhGK*, and *PhGALE*) were markedly downregulated (2.06-fold, 2.23-fold, and 2.61-fold, respectively) compared to those in the control. At 48 h of darkness, two genes (*PhGLA1* and *PhGLA2*) were markedly upregulated (2.63-fold and 4.01-fold, respectively) and two genes (*PhGALE* and *PhGALT*) were markedly downregulated (2.25-fold and 2.25-fold, respectively) compared to those in the control.

To further validate the sequencing data, eight DEGs were randomly selected to detect expression levels by qRT-PCR. The expression level trends for six of eight DEGs were relatively consistent between the qRT-PCR and RNA-sequencing data, indicating the RNA-sequencing results were reliable (Figure S32).

## 3. Discussion

### 3.1. The “Floridean Starch–Floridoside” Metabolic Network of N. haitanensis

In this study, an improved metabolic network of floridean starch–floridoside in *N. haitanensis* was proposed (Figure 3), which better reflected the close connection between these two metabolic pathways. In red algae, the phosphotriose produced by photosynthesis is transported to the cytoplasm and converted to Fru-6-P and DHAP [28]. Under successive reversible catalysis of GPI and PGM, Fru-6-P is converted into Glu-1-P, which then reacts with UTP to generate UDP-Glu by UGP (Figure 3). As a precursor monosaccharide for the synthesis of floridean starch, UDP-Glu can also be transformed to UDP-Gal through GALE and can then synthesize floridoside with Gly-3-P, which is isomerized from DHAP (Figure 3). Thus, the metabolic pathways of floridean starch and floridoside share these three upstream genes (*GPIs*, *PGMs*, and *UGPs*). Gal-1-P, the decomposition product of floridoside and UDP-Glu, can also be reversibly converted to Glu-1-P and UDP-Gal under the catalysis of GALT (Figure 3) [30]. Therefore, Gal-1-P can serve as a precursor for floridean starch biosynthesis by converting to Glu-1-P and then to UDP-Glu. This is similar to the sharp decrease in the radioactivity of the [^14^C] floridoside fraction accompanied by an increase in the labeling of starch and other polysaccharides, indicating that floridoside can be converted into starch and polysaccharides [11,31]. In addition, genomes of most red algae (*C*. *merolae*. *G. sulphuraria*, *P. purpureum*, *N. yezoensis*, *P. umbilicalis*, *C. crispus*, and *G. chorda*) were found to have complete floridean starch and floridoside metabolic pathway genes (Table S1). This suggests that this metabolic network is ubiquitous in unicellular and multicellular red algae, potentially implicating that it played an important role during red algae evolution.

### 3.2. Integration of Genes from Different Sources Contributes to the Floridean Starch and Floridoside Metabolic Pathway Evolution in N. haitanensis

Floridean starch and floridoside metabolic pathway genes in *N. haitanensis* have different origins. Despite previously published detailed phylogenies for *UGP*, *PGM*, *SS*, *BE*, *ISA*, *TPS*, *TPP*, *GALT*, etc. [3,21,30], the phylogenetic relationships of other important genes associated with floridean starch and floridoside metabolic pathways in red algae (*PGM*, *PUL*, *AMY*, *PWD*, *GWD*, *SEX4*, *TPS*, *TPP*, *GALE*, and *GLA*) remain unclear. We, therefore, reconstructed the phylogenetic tree of floridean starch and floridoside metabolic pathway genes and found that 21 genes (or families) of *N. haitanensis* had different origins, including eukaryotic, cyanobacterial, and bacterial origin (Figures S1–S19). Therefore, the “floridean starch–floridoside” metabolic network of *N. haitanensis* showed a mosaic gene origin pattern (Figure 3), similar to algal alginate and fucoidan pathway-related genes [32].

Most gene family members involved in the “floridean starch–floridosid” metabolic network of *N. haitanensis* exhibited the same gene expression profile characteristics following dark treatment, whereas *AMY1* and *AMY2* demonstrated opposing expression profiles following exposure to 24 h and 48 h of darkness (Figure 7). Similarly, the *AMY* gene duplicates of banana and *Camellia sinensis* were differentially expressed after acetylene treatment and cold treatment, respectively [33,34]. In addition, although the *N. haitanensis* genome contains two copies of the *AMY* gene, the genomes of other Bangiaceae species (*N. yezoensis* and *P. umbilicalis*) contain only one. Indeed, gene duplication represents the major factor by which genes confer novel functions that arise during evolution and plays an important role in species’ environmental adaptation [35]. Therefore, the replication of the *PhAMY* gene and the differential expression regulation between *PhAMY* gene duplicates might contribute to the stability of floridean starch content under dark conditions.

Deletion, acquisition, and replication of the starch-binding domain occurred in GWDs, TPS4, and SEX4, respectively. The N terminal of GWD in potato contains an α-amylase domain, which might represent the starch-binding domain of GWD [36]. In red algae, the α-amylase domain was missing in the GWD of *N. haitanensis*, *P. purpureum*, *N. yezoensis*, *P. umbilicalis*, and *G. chorda,* however, was identified in the GWD of *C*. *crispus* and *C*. *merolae* (Figure 2A). Moreover, in PhTPS family proteins, only the N terminal of PhTPS4 contained a CBM20 domain (Figure 1), which is considered to be the starch-binding domain [21]. Meanwhile, multiple copies of CMB20 domains were found in SEX4 of ciliates, cryptophytes, and red algae [37]. We also observed that the number of CBM20 domains varied from one to three in red algae species (Figure 2B); however, these differences did not reflect the differences between unicellular (Cyanidiophyceae and Porphyridiophyceae) and multicellular red algae groups (Bangiophyceae and Florideophyceae), nor did they reflect differences among species within a family (Bangiophyceae). Therefore, it can be speculated that alterations in the number of SEX4 CBM20 domains in red algae are an independent evolutionary event. Furthermore, the sequence of the three CBM20 domains of PhSEX4 showed amino acid diversity, similar to that observed in *Oxytrichia trifallax* (Ciliophora), which lacks certain residues necessary for its carbohydrate-binding function [37]. These results indicate that the floridean starch and floridoside metabolic pathway genes of *N. haitanensis* underwent acquisition, deletion, and duplication of conserved domains during their evolution.

### 3.3. Floridean Starch and Floridoside Content in N. haitanensis Vary with Different Trends under Continuous Darkness

Floridoside and isofloridoside are two enantiomeric forms of α-D-galactosyl-glycerol [19] and are both synthesized by the condensation reaction of Gly-3-P and UDP-Gal [21]. Although Sun et al. [21] found that both TPS1 and TPS4 are involved in floridoside and isofloridoside biosynthesis, no evidence has been presented to confirm whether the enzymes involved in their degradation are distinguishable. Therefore, to better understand the floridoside metabolic pathway in *N. haitanensis*, the gene expression and metabolite content of floridoside and isofloridoside metabolism pathways were analyzed together (Figure 5, Figure 7 and Figure S33).

Floridoside content decreased continuously under continuous darkness (for 24 h and 48 h). Compared with that in the control group (6 h of light), after 6 h in the dark, there was no significant change in floridoside content, whereas isofloridoside content increased, and their degradation product (Gal-1-P) contents showed no significant changes (Figure 5); overall, the total floridoside and isofloridoside contents increased significantly (Figure S34). At the transcript level, *PhTPP2*, which catalyzes the dephosphorylation of (iso)floridoside phosphate, and *PhGLA2*, which catalyzes the degradation of (iso)floridoside, were significantly upregulated at 6 h in the dark (Figure 7). With an increase in darkness duration (24 h and 48 h), floridoside and Gal-1-P contents decreased compared to those in the control, and isofloridoside content returned to the control level; overall, the total floridoside and isofloridoside contents decreased significantly (Figure S34 and Figure 5). At the transcript level, *PhGLA1* and *PhGLA2*, which catalyze the degradation of (iso)floridoside, were significantly upregulated (Figure 7). This indicates that (iso)floridoside metabolism in *N. haitanensis* was dominated by biosynthesis under short-term dark conditions and degradation under continuous dark conditions, which was similar to a previous finding that reported that the total floridoside and isofloridoside contents in *P. purpurea* decreased after exposure to constant darkness for 24 h [10]. Moreover, the expression of floridoside catabolism pathway genes was upregulated, resulting in a significant decrease in *N. haitanensis* floridoside content under continuous dark conditions. Similar to our results, the content of floridoside in *P*. *purpureum* (dark 24 h) was found to significantly decrease under dark conditions [11].

The staining results and the starch content analysis showed that the content of floridean starch in *N. haitanensis* remained stable under continuous dark conditions. *PhUGP*, which transforms Glu-1-P into UDP-Glu and provides substrates for floridean starch synthesis, as well as the floridean starch synthase gene (*PhSS_UDPG_*), were upregulated under dark conditions (for 6 h, 24 h, and 48 h) (Figure 7). These results may indicate that in *N. haitanensis*, floridean starch continues to be synthesized in the dark, similar to mannitol synthesis in *Saccharina japonica* (Ochrophyta, Phaeophyceae) [38]. Moreover, certain genes involved in floridean starch degradation were upregulated (*PhGWD2* and *PhAMY1*), while others were downregulated (*PhAMY2*, *PhISA*, and *PhBAM*; Figure 7), indicating that floridean starch catabolism genes have different regulatory patterns under dark conditions. The content of maltose, the degradation product of floridean starch, increased by 55.36% in 6 h of darkness, and returned to the control level after 24 and 48 h of darkness (Figure 5). This differed from the patterns observed in land plants. For example, in dark conditions, starch is almost completely degraded in *Arabidopsis thaliana* and *Phalaenopsis* “Edessa”, whereas maltose content rapidly increases [39,40]. This indicates that floridean starch content remained unchanged in dark conditions, which results from a balance between floridean starch synthesis and degradation. The cells of *P*. *purpureum* had an abundance of floridean starch, even after one week in the dark, which was similar to our results [8]. In contrast, floridean starch content was markedly decreased in *G. lemaneiformis* and *Corallina maxima* (formerly, *Serraticardia maxima*) (Rhodophyta) [9,41], indicating that the expression regulation of starch metabolism pathways may differ among different species and different generations. This unique metabolic characteristic of floridean starch in *N. haitanensis* may represent a valuable mechanism for adjusting to environmental changes. However, this specific mechanism needs to be further explored.

## 4. Materials and Methods

### 4.1. Algal Materials and Culture Conditions

*N. haitanensis* thalli were collected from natural populations on Dongjia Island, Fujian Province, China, on 11 December 2019, and transferred to an indoor aerated culture system in Provasoli’s enrichment seawater (PES) medium at 21 °C in darkness for a 48 h recovery culture. Afterwards, the algae were cultured at 21 °C under 50 μmol m^−2^s^−1^ irradiance (12 h light/dark photoperiod). The growth medium was refreshed every two days. Then, the thalli of same size were selected and divided into four groups: one control group (C) and three groups for darkness treatments. The four groups were cultured in the same volume of PES medium at 21 °C. After 12 h of light culture (50 μmol m^−2^s^−1^), the three dark-treated algae groups were exposed to darkness for 6 h (T1), 24 h (T2), and 48 h (T3), respectively. The control group was cultured under light (50 μmol m^−2^s^−1^) for 6 h. Each group contained three biological replicates, and each biological replicate was collected randomly and stored at −80 °C until transcriptome sequencing and metabolite detection.

### 4.2. Photochemical Reactions

The photosynthetic properties of the thalli were measured using a Modulated Imaging-PAM fluorometer (Heinz Walz, Effeltrich, Germany). Three parameters, F_0_, Fv/Fm, and YII, were used. The minimal (F_0_) and maximal chlorophyll fluorescence (F_m_) values were determined after 15 min of dark adaptation; from these, the maximal photosystem II (PSII) quantum yield was calculated (F_v_/F_m_ = (F_m_−F_0_)/F_m_) [42]. Actinic illumination was then switched on and saturating pulses were applied to determine light-acclimated maximal fluorescence (F_m_^’^) and steady-state fluorescence yield (F_s_). The quantum yield of photochemical energy conversion in PSII (YII = (F_m_^’^−F_s_)/F_m_^’^) was calculated [43].

### 4.3. Metabolite Detection

#### 4.3.1. Floridean Starch

The thalli samples were oven dried and pulverized. The 100 mg sample was homogenized in 4 mL of 80% ethanol, stored at 70 °C for 2 h and centrifuged at 12,000× *g* for 10 min to collect the residues. Four milliliters of 80% ethanol were added to the residue and centrifuged at 12,000× *g* for 10 min; this process was repeated three times. Subsequently, 3 mL of perchloric acid was added to the residue, agitated on a vortex at 1500 rpm for 2 min, further mixed using a rotary shaker at 200 rpm for 10 min, and left to react for 5 min. The extract (0.4 mL) was then mixed with 1.6 mL of anthrone reagent and heated at 95 °C for 10 min. The absorbance A620 was measured with a glucose standard and the floridean starch content was calculated by multiplying glucose concentration by a conversion factor of 0.9 [44].

#### 4.3.2. Floridoside and Isofloridoside

Frozen samples were ground to powder in liquid nitrogen, 100 mg of which was then placed in a centrifuge tube with 700 μL of 80% methanol and placed on ice for 1 h to facilitate floridoside and isofloridoside extraction. After centrifugation at 15,000× *g* for 10 min, the supernatant was collected. The above steps were repeated an additional time, and the extracted supernatants were mixed. The sample extracts were analyzed using a Waters HPLC system at 25 °C with a Q Exactive high-resolution mass spectrometer (Thermo Scientific, Waltham, MA, USA) by Sanshu Bio-tech, Ltd. (Nantong, China). The analytical conditions were as follows: HPLC column, Waters BEH Amide (50 × 2.1 mm, 1.7 μm); solvent system A: H_2_O; solvent system B: 5 mM NH_4_AC; injection volume: 2 μL. The mass spectrometric conditions were as follows: sheath gas pressure flow rate, 40 L/min; auxiliary gas pressure flow rate, 10 Abs; spray voltage, −2800 V; vaporizer temperature, 350 °C; capillary temperature, 320 °C. Floridoside content of the samples was calculated by comparing their peak area with that of the floridoside standard (Glycosci). As the commercial standard of isofloridoside was unavailable, isofloridoside was qualitatively determined by mass spectrometry, compared with literature data [21], and quantified using the calibration curve of the floridoside standard.

#### 4.3.3. Soluble Sugars (Glucose and Maltose)

Frozen samples were ground to powder in liquid nitrogen. A powdered sample (100 mg) was placed in a centrifuge tube, to which 700 μL of 80% methanol was added and placed at 50 °C for 2 h for extraction. The samples were diluted with 700 μL of H_2_O and centrifuged at 10,000× *g* for 3 min. The supernatant was collected, 700 μL of CHCl_3_ was added, and the mixture was centrifuged at 10,000× *g* for 3 min. The supernatant was then analyzed using a Thermo ICS5000 HPLC system (Dionex, Thermo Scientific, Waltham, MA, USA) by Sanshu Bio-tech, Ltd. (Nantong, China). The analytical conditions were as follows: HPLC column, CarboPac™ PA200 (250 × 3.0 mm, 5.5 µm); solvent system A: H_2_O; solvent system B: 200 mM NaOH; injection volume: 25 μL; column temperature: 30 °C. Sugar contents were calculated based on the sample and standard peak areas.

#### 4.3.4. Phosphorylated Sugars (Fructose-6-Phosphate, Glucose-6-Phosphate, Glucose-1-Phosphate, and Galactose 1-Phosphate), UDP Sugars (UDP-Galactose and UDP-Glucose), and Glycerol-3-Phosphate

Frozen samples were ground to powder in liquid nitrogen. A powdered sample (100 mg) was placed in a centrifuge tube to which 1.2 mL of 50% methanol was added, and the tube was placed at 40 °C for 1 h for extraction. The samples were centrifuged at 15,000× *g* for 10 min. The supernatant was collected, 700 μL CHCl_3_ was added, and the mixture was centrifuged at 10,000× *g* for 3 min. The supernatant was then analyzed using a Thermo ICS5000 HPLC system (Thermo Fisher Scientific) by Sanshu Bio-tech, Ltd. (Nantong, China). The analytical conditions were as follows: HPLC column, CarboPac™ PA10 (250 × 4.0 mm, 10 μm); solvent system A: 10 mM NaOH; solvent system B: 10 mM NaOH, 50 mM NaAC; injection volume: 20 μL; column temperature: 30 °C. The content of each metabolite was calculated based on the sample and standard peak areas.

### 4.4. Staining of Starch Granules

*N. haitanensis* thalli were fixed in 4% paraformaldehyde for 24 h, decolored in 70% ethanol for an additional 24 h, and stained with a solution of 1% (*w*/*v*) I_2_ and 2.5% KI for 30 min [45]. The entire staining procedure was performed at 21 °C.

### 4.5. Gene Identification

Genes were identified by analyzing genomic (PRJNA503796) data for *Neoporphyra haitanensis*, as well as the genome and protein data of other red algae (*Chondrus crispus*, *Cyanidioschyzon merolae*, *Gracilariopsis chorda*, *Galdieria sulphuraria*, *Porphyrideum purpureum*, *Neopyropia yezoensis*, and *Porphyra umbilicalis*) downloaded from NCBI (https://www.ncbi.nlm.nih.gov/, accessed on 10 September 2019) and OneKP (https://www.onekp.com, accessed on 12 September 2019). BLAST analysis was performed to search for homologous genes in red algae by using known proteins involved in floridean starch and floridoside metabolic pathways from NCBI, MGU (https://marinegenomics.oist.jp/algae/gallery, accessed on 20 September 2019), and Ensembl (http://plants.ensembl.org/index.html, accessed on 20 September 2019) as queries with an E-value < 1 × 10^−5^. All candidate genes were further screened by examining the existence of a conserved domain by using the Conserved domain (CDD) program (https://www.ncbi.nlm.nih.gov/cdd/?term=, accessed on 10 October 2019). All downloaded and identified sequences are listed in Table S3.

Twenty-one genes (or families) were used for further analysis of the *N. haitanensis* genome after comprehensive examination. Sequence lengths, molecular weights, isoelectric points, and instability indices of identified proteins were determined using the online ProtParam tool (SIB Swiss Institute of Bioinformatics, Geneva, Switzerland; http://web.expasy.org/protparam, accessed on 23 June 2020). The genomic DNA and full-length cDNA sequences were aligned using the TBtools (v1.098669) software (South China Agricultural Uinversity, Guangzhou, China) to determine the intron–exon structure and the number of introns [46]. The cis-acting regulatory elements were predicted using the PlantCARE database (http://bioinformatics.psb.ugent.be/webtools/plantcare/html/, accessed on 23 June 2020) and mapped using the TBtools (v1.098669) software (South China Agricultural Uinversity, Guangzhou, China). Multiple-sequence alignment was conducted using MEGA X with default parameters [47]. GeneDoc (v2.7) software (Pittsburgh Supercomputing Center, Pittsburgh, PA, USA; https://github.com/karlnicholas/GeneDoc, accessed on 23 June 2021) was used for homology shading and scoring among the aligned sequences.

### 4.6. Phylogenetic Analysis

Phylogenetic trees were constructed based on full-length amino acid sequences by using MrBayes (v3.2) software (MrBayes: Bayesian Inference of Phylogeny, http://mrbayes.sourceforge.net accessed on 23 October 2020) [48]. The MCMC analysis was conducted (prset Aamodelpr = mixed) as follows: the chains were run for over 10,000,000 generations, and trees were sampled every 1000 generations until the average standard deviations of split frequencies were below 0.01. The first 25% of the samples were discarded, and the remaining samples were constructed using a consensus tree. The resulting phylogenetic tree was constructed using FigTree (v1.4.4) (Andrew Rambaut Institute of Evolutionary Biology, University of Edinburgh, Edinburgh, UK; http://tree.bio.ed.ac.uk/software/figtree, accessed on 23 October 2020). All protein accession numbers are listed in Table S3.

### 4.7. RNA Extraction, Library Construction, and Illumina Sequencing

Total RNA was extracted from the cells by using TRIzol® Reagent (Invitrogen, Carlsbad, CA, USA) according to the manufacturer’s instructions, and genomic DNA was removed using DNase I (TaKara, Dalian, China). RNA-seq transcriptome libraries were prepared using the TruSeqTM RNA sample preparation Kit from Illumina (San Diego, CA, USA), and end repair, A-base addition, and ligation of the Illumina-indexed adaptors were performed according to the Illumina protocol. After quantification by TBS380, paired-end libraries were sequenced by Illumina NovaSeq 6000 (150 bp × 2, Shanghai BIOZERON Co., Ltd, Shanghai, China). Raw paired-end reads were trimmed, and quality controlled using Trimmomatic with the parameters SLIDINGWINDOW:4:15 MINLEN:75 [49]. The clean reads were then separately aligned to the reference genome with the orientation mode using Hisat2 software (Johns Hopkins University, Baltimore, MD, USA); https://daehwankimlab.github.io/hisat2/, accessed on 23 March 2020) to map the data with default parameters. Quality assessment of the data was performed using Qualimap (v2.2.1) (Max Planck Institute for Infection Biology, Berlin, Germany; Bioinformatics Department of Centro de Investigación Príncipe Felipe (CIPF), Valencia, Spain) [50]. Htseq (v0.11.271.) (Stanford University, CA, USA; Heidelberg University, Baden-Württemberg, Germany; https://htseq.readthedocs.io/en/release_0.11.1/, accessed on 8 April 2020) was used to count each gene read.

### 4.8. Differential Expression Analysis and Functional Enrichment

To identify DEGs between the samples, the expression level of each gene was calculated using the fragments per kilobase of exon per million mapped reads (FPKM) method. The R statistical package edgeR (http://www.bioconductor.org/packages/release/bioc/html/edgeR.html/, accessed on 15 April 2020) was used for differential expression analysis. The DEGs between two samples were selected based on a logarithmic fold change >2 and a false discovery rate (FDR) <0.05. To understand the functions of the DEGs, Gene ontology (GO) functional enrichment and Kyoto Encyclopedia of Genes and Genomes (KEGG) pathway analyses were performed using Goatools (https://github.com/tanghaibao/Goatools, accessed on 20 April 2020) and KOBAS (http://kobas.cbi.pku.edu.cn/home.do, accessed on 20 April 2020). DEGs were considered significantly enriched in GO terms and metabolic pathways when their Bonferroni-corrected *p*-value was <0.05.

### 4.9. qRT-PCR Analysis

Among DEGs, eight genes were randomly selected for qPCR validation. The qRT-PCR was performed with a Roche LightCycler 480 PCR Detection System (Roche, Germany). The analysis of each sample was repeated three times, and the 2^−ΔΔCT^ method was used to perform data analysis. The *N. haitanensis* ubiquitin-conjugating enzyme gene (*PhUBC*) was used as an internal reference gene [51]. In this study, all the primers are shown in Table S14.

### 4.10. Statistical Analysis

One-way analysis of variance (ANOVA) with Tukey’s test was used to compare the differences between groups by using SPSS software (version 26.0; IBM, Armonk, NY, USA). Data are presented as means ± standard errors (SE).

## 5. Conclusions

In summary, the “floridean starch–floridoside” metabolic network in *N. haitanensis* was characterized in the present study. Our phylogenetic analysis demonstrated that the genes in this metabolic network exhibited a mosaic origin pattern. Duplication of the *AMY* gene and the CBM20 domain of SEX4 indicated the evolutionary distinctiveness of *N. haitanensis*. Our transcriptomic analysis and metabolite determination showed that the “floridean starch–floridoside” metabolism in *N. haitanensis* is a dynamic process under continuous dark conditions, in which floridoside is consumed continuously, whereas floridean starch content remains stable to maintain carbon storage in *N. haitanensis*. This unique metabolic characteristic of floridean starch in *N. haitanensis* may represent a valuable mechanism for adjusting to environmental changes. Finally, our results provided a theoretical basis for elucidating the biosynthesis and catabolism of floridean starch and floridoside in *N. haitanensis*. Moreover, we provided a series of candidate genes for the synthesis of floridean starch and floridoside in vitro. In the future, in-depth studies of carbohydrate metabolic pathways and their regulation would be helpful for understanding the differences between algae and land plants in terms of carbon utilization and the mechanism of carbohydrate-based regulation for adapting to environmental changes.

## Figures and Tables

**Table 1:** Features of floridean starch and floridoside metabolic pathway genes in *Neoporphyra haitanensis*.

Gene Name	Gene ID	Amino Acids	Open Reading Frame (bp)	Molecular Weight (kDa)	Isoelectric Point	Number of Introns	Instability Index
*PhGPI1*	ph06855.t1	559	1680	59.63993	5.86	1	28.18
*PhGPI2*	ph10696.t1	638	1917	70.23072	5.81	0	36.16
*PhPGM1*	ph07345.t1	524	1575	53.45336	4.64	2	31.59
*PhPGM2*	ph08542.t1	430	1293	43.42822	5.43	1	31.2
*PhPGM3*	ph01308.t1	586	1761	61.88904	5.1	1	27.5
*PhUGP1*	ph10255.t1	504	1515	55.60583	6.19	0	28.03
*PhUGP2*	ph10249.t1	510	1533	56.19654	6.34	0	27.52
*PhSS_UDPG_*	ph07441.t1	1767	5304	189.20083	6.04	1	44.02
*PhISA*	ph06437.t1	786	2361	84.18759	5.64	0	33.81
*PhBE*	ph11470.t1	755	2268	85.18558	5.92	2	37.93
*PhPUL*	ph02603.t1	1779	5340	180.68102	5.5	1	41.86
*PhAMY1*	ph05661.t1	447	1344	47.39023	8.72	2	36.3
*PhAMY2*	ph09788.t1	505	1518	54.85313	5.67	1	29.27
*PhBAM*	ph06397.t1	458	1377	49.31007	5.57	0	39.7
*PhGWD1*	ph05026.t1	1344	4035	144.2512	5.33	0	39.64
*PhGWD2*	ph09623.t1	1018	3057	101.82198	6.52	2	33.75
*PhPWD*	ph11079.t1	1039	3120	103.82096	5.49	1	36.37
*PhPHO*	ph09474.t1	883	2652	99.18552	6.1	1	36.27
*PhDPE2*	ph04961.t1	1014	3045	111.16318	5.12	0	41.86
*PhGALE*	ph04371.t1	344	1035	37.82118	5.26	0	36.52
*PhSex4*	ph06018.t1	739	2220	75.19834	5.45	0	47.62
*PhGPDH_m_*	ph01923.t1	711	2136	75.27303	8.58	1	33.98
*PhGPDH_c_1*	ph00687.t1	405	1218	40.98797	7.61	1	41.81
*PhGPDH_c_2*	ph05255.t1	386	1161	41.85018	5.52	0	27.84
*PhGK*	ph03288.t1	576	1731	60.3038	4.79	1	34.82
*PhTPS1*	ph10158.t1	1153	3462	124.15916	6.73	0	46.18
*PhTPS2*	ph10797.t1	1505	4518	159.36145	5.93	0	52.21
*PhTPS4*	ph07423.t1	1007	3024	112.57108	5.79	1	34.78
*PhTPP1*	ph05156.t1	454	1365	46.84337	5.04	1	39.21
*PhTPP2*	ph05159.t1	266	801	28.07624	5.31	1	39.98
*PhGLA1*	ph02625.t1	1109	3330	120.26068	5.98	7	47.35
*PhGLA2*	ph00656.t1	1098	3297	114.41963	6.06	1	42.92
*PhGLA3*	ph08573.t1	1098	3297	119.0251	5.83	3	44.03
*PhGLA4*	ph05433.t1	542	1629	53.53138	9.4	1	28.14
*PhGALT*	ph04880.t1	364	1095	41.01006	5.44	0	38.06

## Data Availability

The transcriptome sequencing data for this study can be found in the NCBI Sequence Read Archive under the BioProject: PRJNA670264 (https://dataview.ncbi.nlm.nih.gov/object/PRJNA670264?reviewer=6gc42o6ms9tsktj8srehbqkb63, accessed on 10 September 2019).

## Supplementary Materials

The following are available online at https://www.mdpi.com/article/10.3390/md19120664/s1, Figures S1–S19: Bayesian phylogenetic trees of floridean starch and floridoside metabolic pathway genes, Figure S20: Diagram of gene coverage analysis results, Figure S21: Sequencing quality detection, Figure S22: Volcano plots of differentially expressed genes (DEGs), Figures S23–S25: Gene ontology (GO) functional annotation of differentially expressed genes (DEGs), Figures S26–S28: Gene ontology (GO) functional enrichment of differentially expressed genes (DEGs), Figures S29–S31: Pathway functional enrichment analysis of differentially expressed genes (DEGs), Figure S32: qRT-PCR validation of RNA-seq at 8 selected target genes, Figure S33: LC-MS analysis of floridoside and isofloridoside, Figure S34: The total floridoside and isofloridoside contents, Table S1: Floridean starch and floridoside metabolism, Table S2: Sequences of floridean starch and floridoside metabolic pathway genes in *Neoporphyra haitanensis*, Table S3: List of sequences used in this study and their corresponding accession numbers, Table S4: Summary of quality preprocessing of RNA sequencing data, Table S5: Mapping ratio statistics, Table S6–S8: Gene Ontology (GO) functional annotation for differentially expressed genes (DEGs), Table S9: Gene Ontology (GO) enriched data for differentially expressed genes (DEGs), Table S10–S12: Kyoto Encyclopedia of Genes and Genomes (KEGG) enriched data for differentially expressed genes (DEGs), Table S13: Expression levels of genes involved in floridean starch and floridoside metabolism in each sample, Table S14: Sequences of the primers used in this study.
