# Supplementary material for: Floridean Starch and Floridoside Metabolic Pathways of Neoporphyra haitanensis and Their Regulatory Mechanism under Continuous Darkness
Source: Mar Drugs. 2021 Nov 26;19(12):664. doi: 10.3390/md19120664 (PMC8703398; doi:10.3390/md19120664)
Supplement: Supplementary file 1 [file marinedrugs-19-00664-s001.zip › Supplementary_Figures.pdf]

Supplementary Material

Floridean starch and floridoside metabolic pathways of *Neoporphyra haitanensis* and their regulatory mechanism under continuous darkness

Yahui Yu, Xuli Jia, Wenlei Wang, Yuemei Jin, Weizhi Liu, Dongmei Wang, Yunxiang Mao, Chaotian Xie, Tao Liu

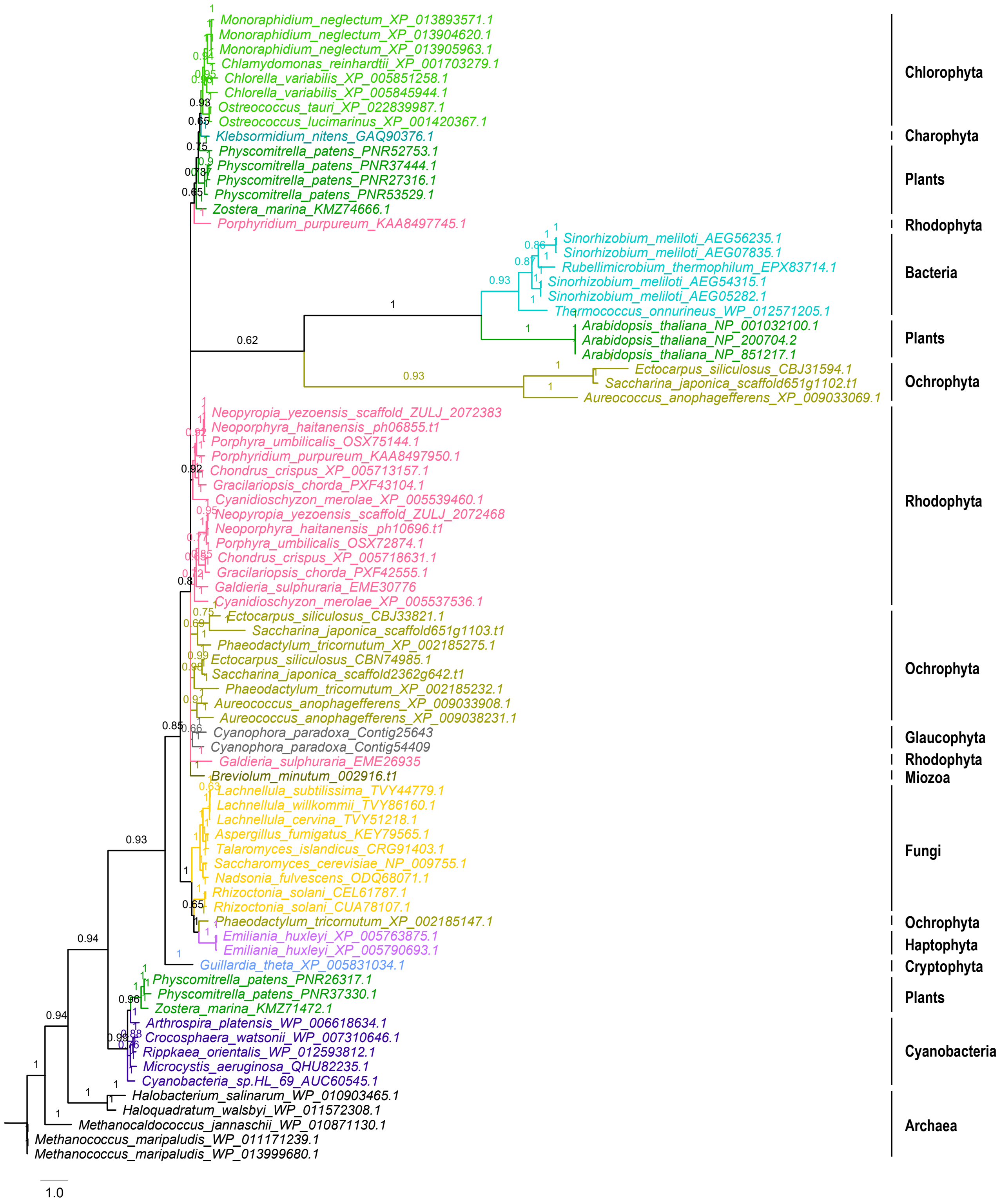

Figure S1. Bayesian phylogenetic tree of glucose-6-phosphate isomerase (GPI) based on GPI amino acid sequences. The GPIs of Neoporphyra haitanensis originated from primary endosymbiotic eukaryotic hosts. All protein accession numbers are listed in Table S3.

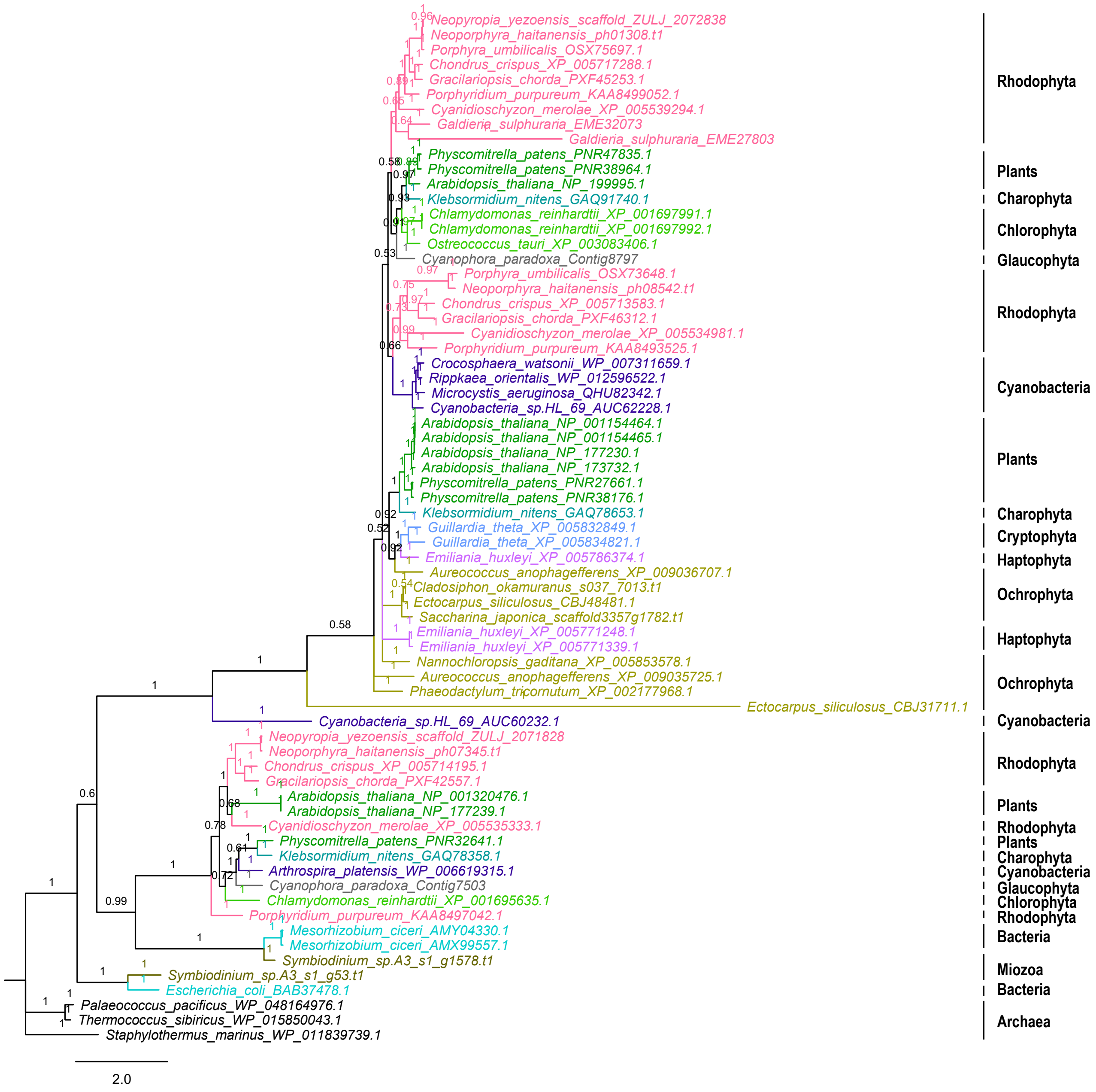

Figure S2. Bayesian phylogenetic tree of phosphoglucomutase (PGM) based on PGM amino acid sequences. The PGMs of *Neoporphyra haitanensis* seemed that there were two origins, one was primary endosymbiotic cyanobacteria and the other cannot be confirmed. All protein accession numbers are listed in Table S3.

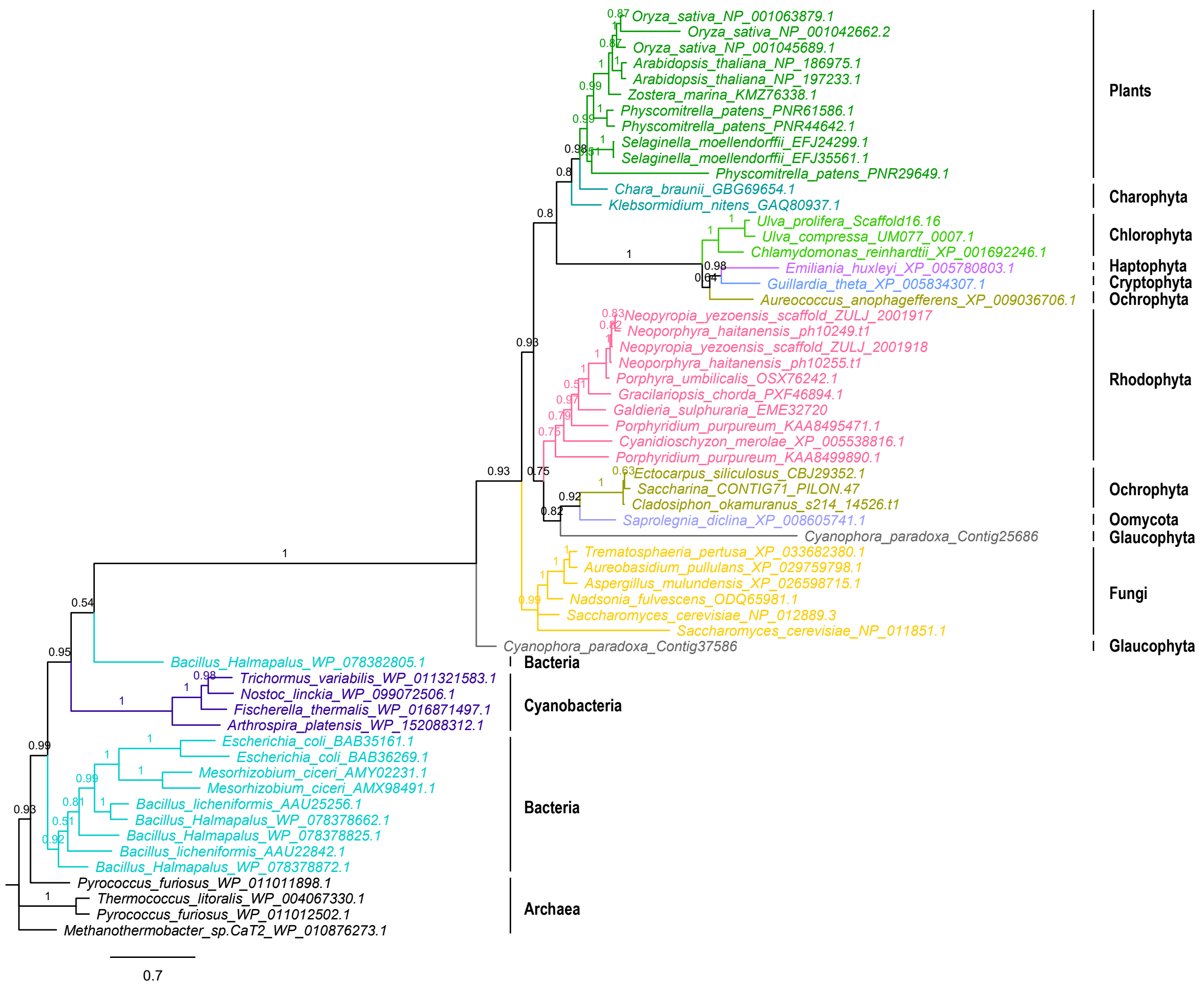

Figure S3. Bayesian phylogenetic tree of UDP-glucose pyrophosphorylase (UGP) based on UGP amino acid sequences. The UGPs of *Neoporphyra haitanensis* originated from primary endosymbiotic eukaryotic hosts. All protein accession numbers are listed in Table S3.

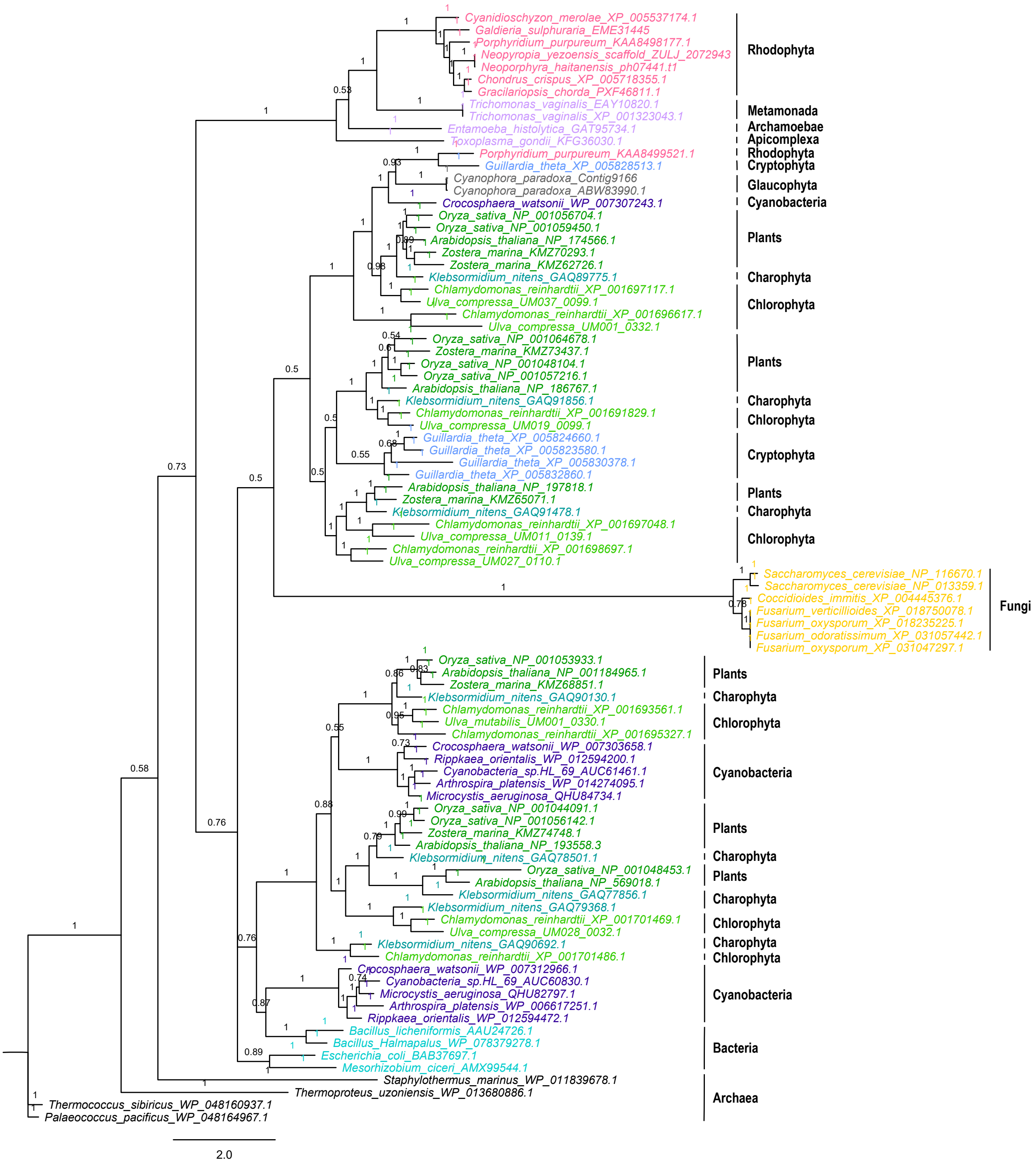

Figure S4. Bayesian phylogenetic tree of glycogen (starch) synthases (G(S)S) based on G(S)S amino acid sequences. The SSUDPG of *Neoporphyra haitanensis* may have originated from primary endosymbiotic eukaryotic hosts. All protein accession numbers are listed in Table S3.

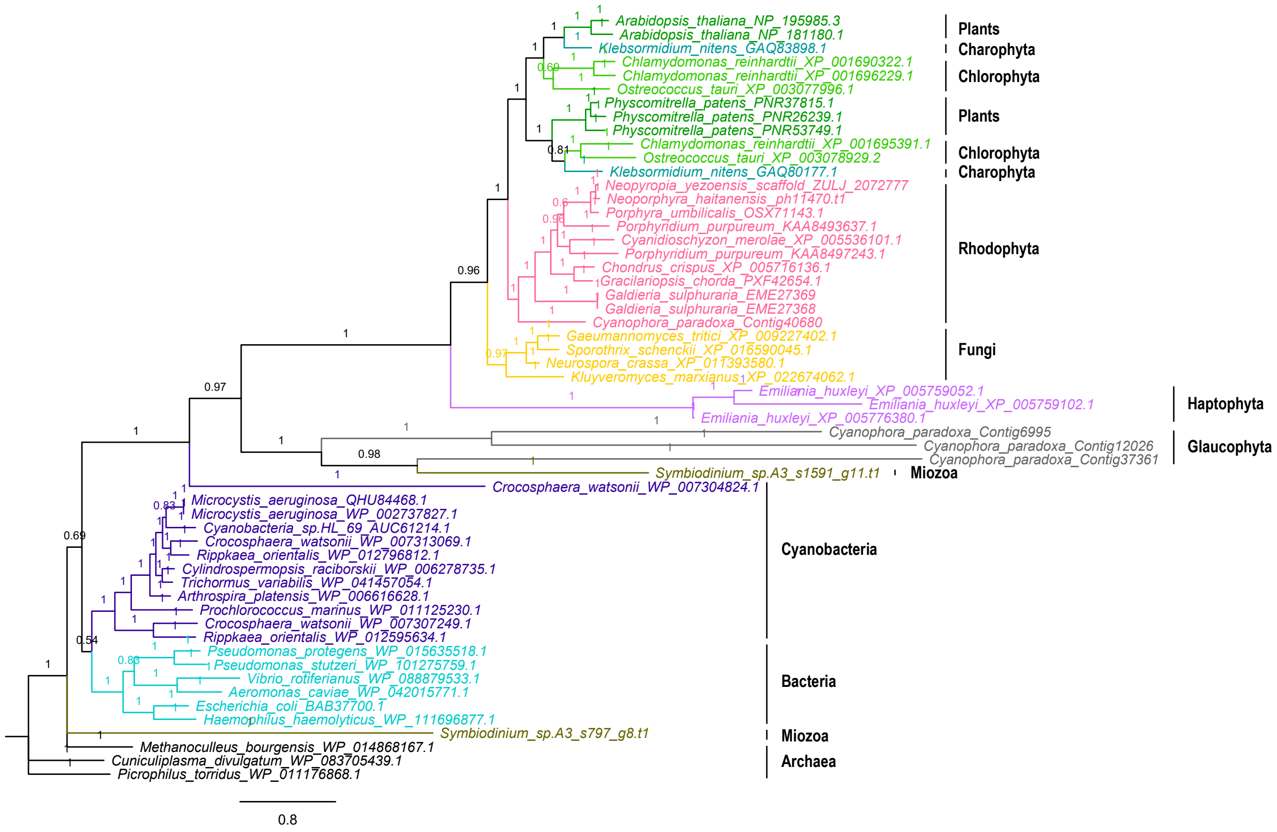

Figure S5. Bayesian phylogenetic tree of branching enzyme (BE) based on BE amino acid sequences. The BE of *Neoporphyra haitanensis* originated from primary endosymbiotic eukaryotic hosts. All protein accession numbers are listed in Table S3.

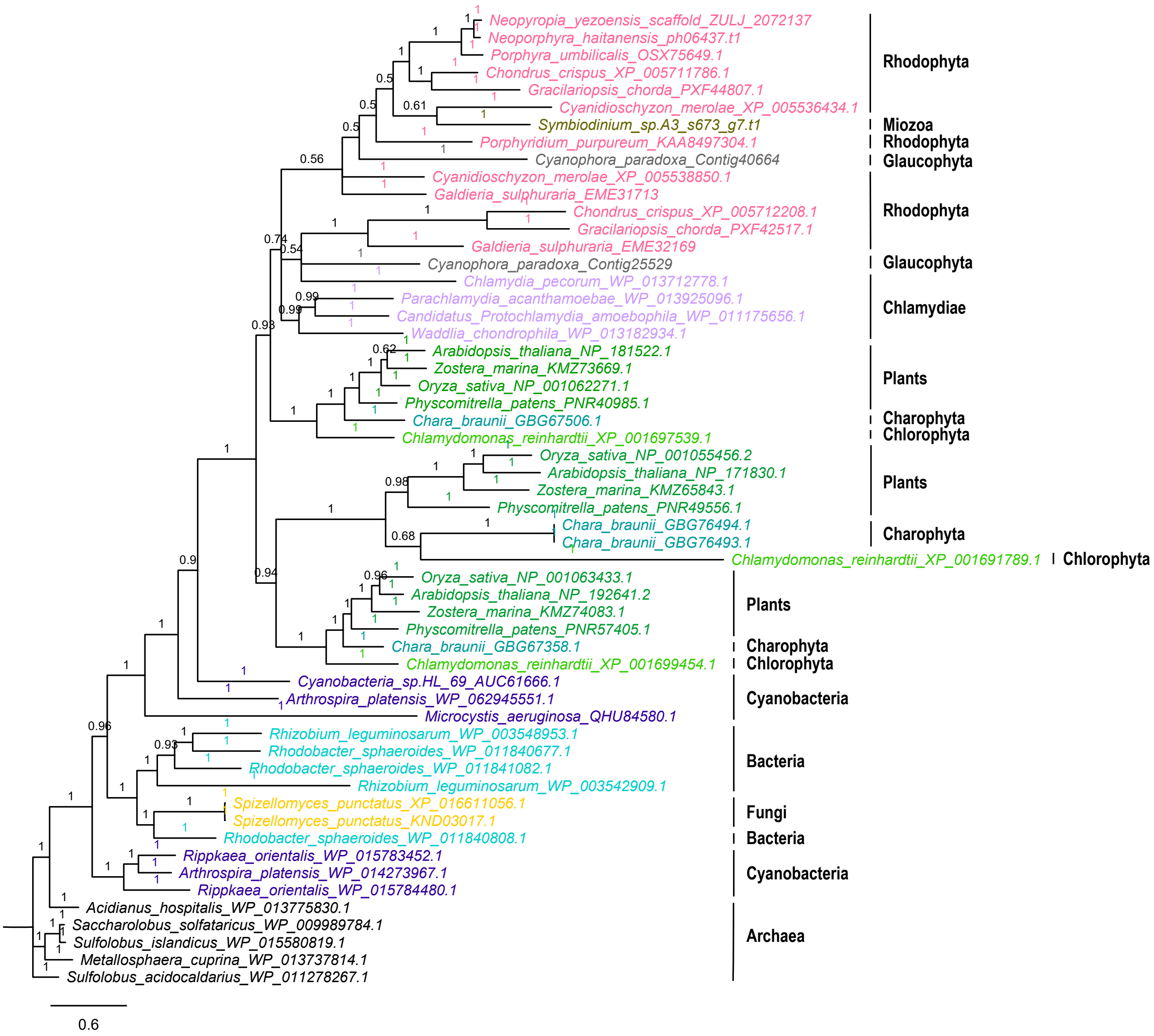

Figure S6. Bayesian phylogenetic tree of isoamylase (ISA) and glycogen debranching enzyme (GlgX) based on ISA and GlgX amino acid sequences. The ISA of *Neoporphyra haitanensis* may have originated from bacteria via horizontal gene transfer. All protein accession numbers are listed in Table S3.

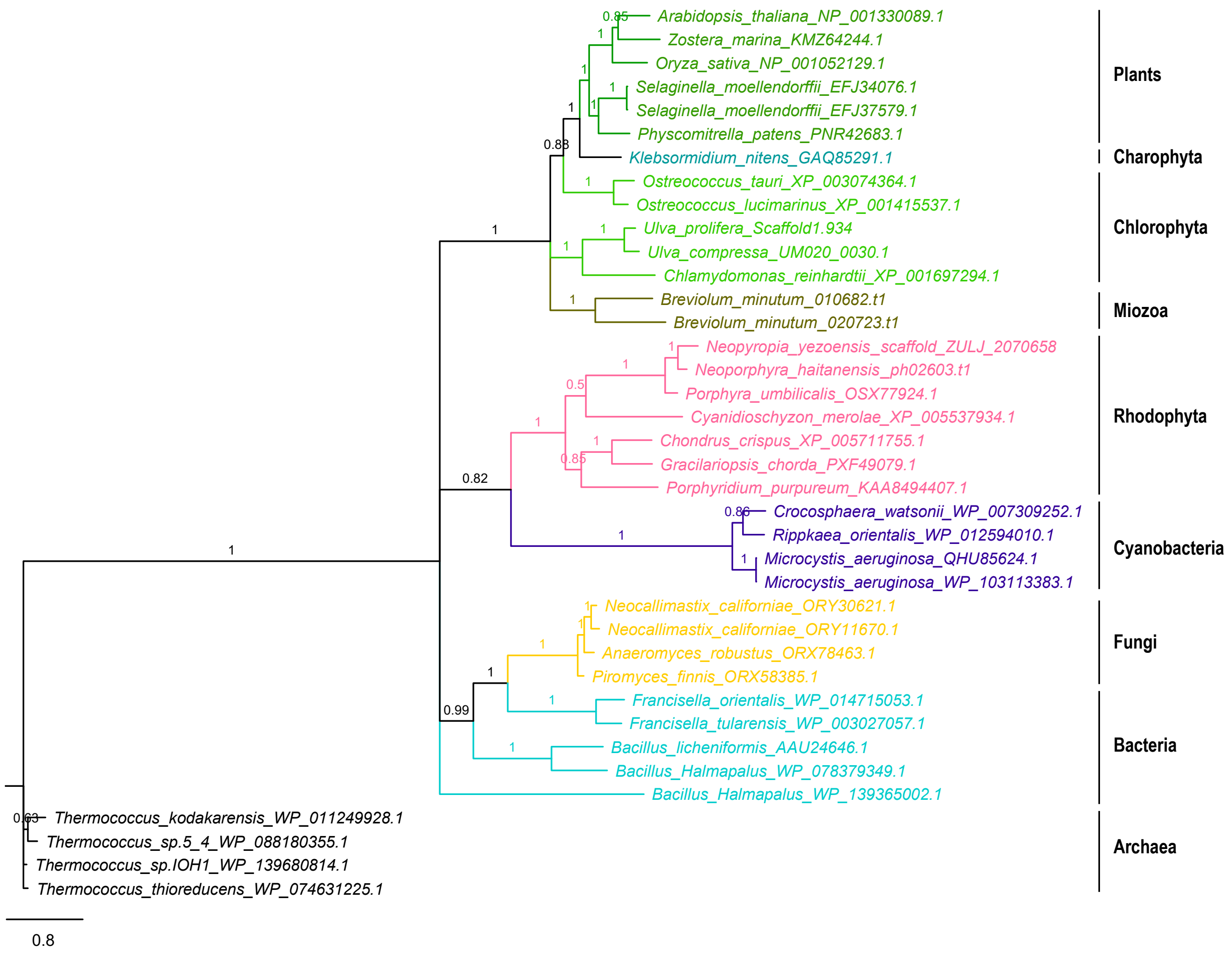

Figure S7. Bayesian phylogenetic tree of pullulanase (PUL) based on PUL amino acid sequences. The PUL of *Neoporphyra haitanensis* originated from primary endosymbiotic cyanobacteria. All protein accession numbers are listed in Table S3.

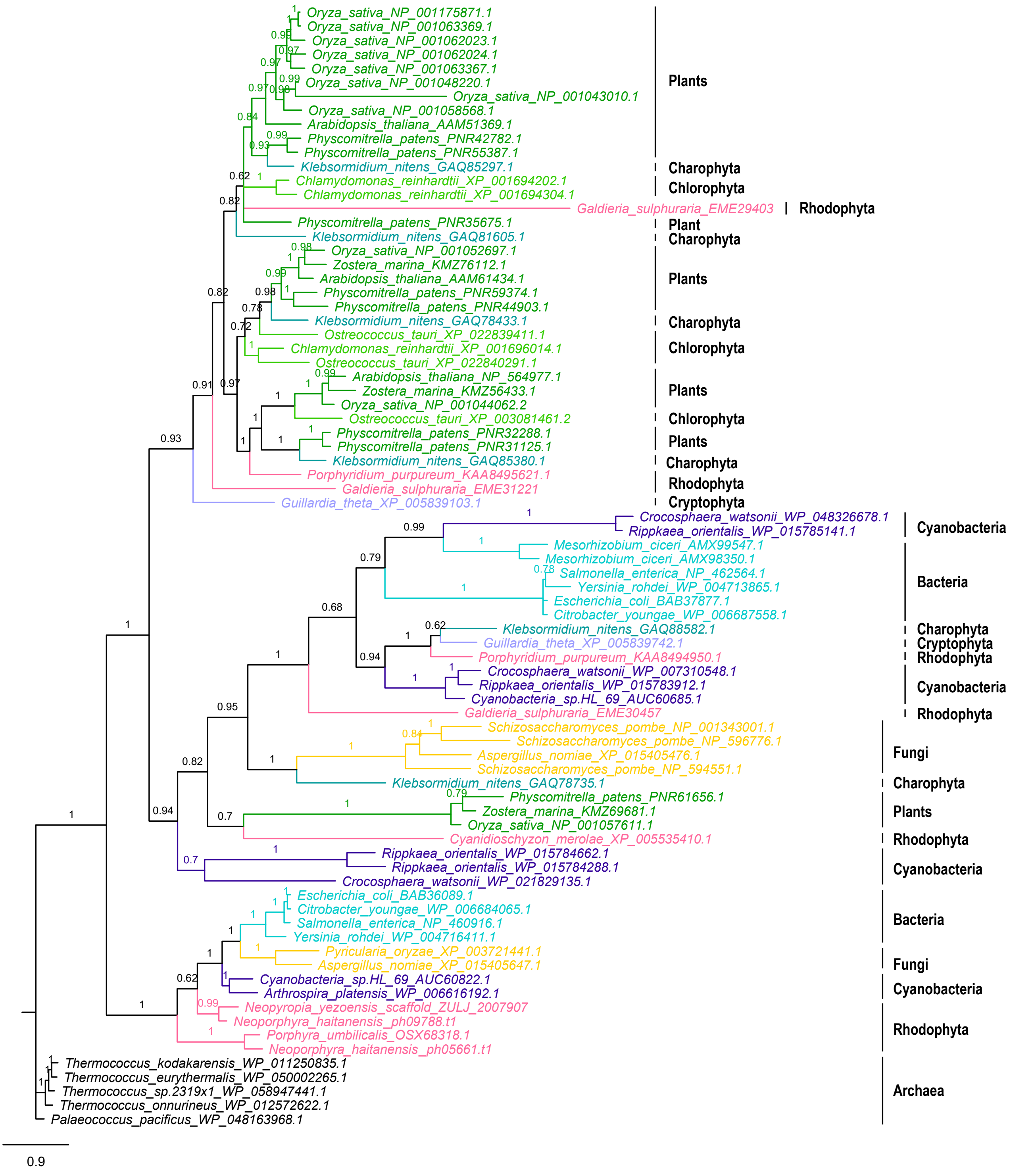

Figure S8. Bayesian phylogenetic tree of alpha-amylase (AMY) based on AMY amino acid sequences. The AMYs of *Neoporphyra haitanensis* may have originated from primary endosymbiotic eukaryotic hosts. All protein accession numbers are listed in Table S3.

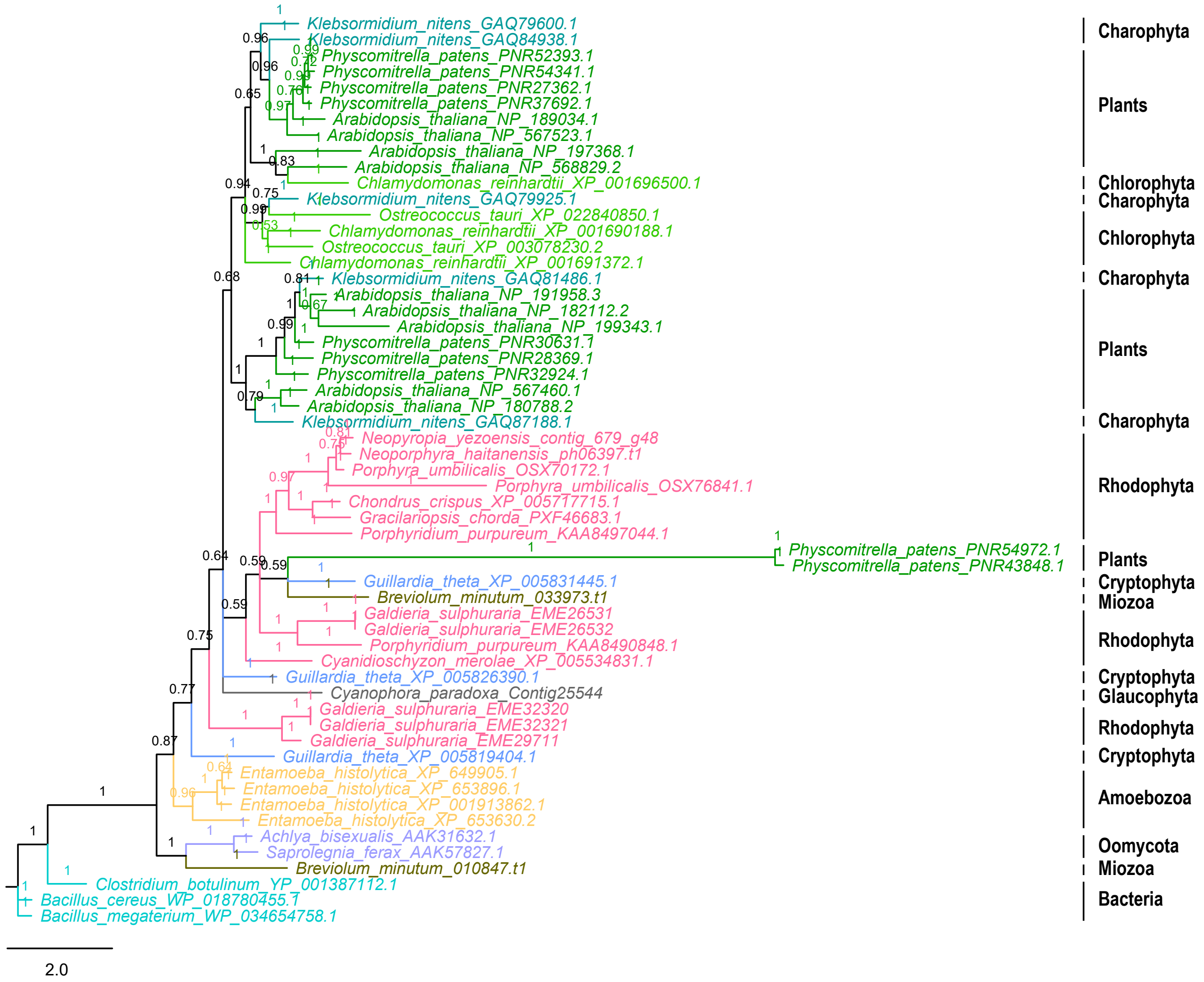

Figure S9. Bayesian phylogenetic tree of beta-amylase (BAM) based on BAM amino acid sequences. The BAM of *Neoporphyra haitanensis* may have originated from primary endosymbiotic eukaryotic hosts. All protein accession numbers are listed in Table S3.

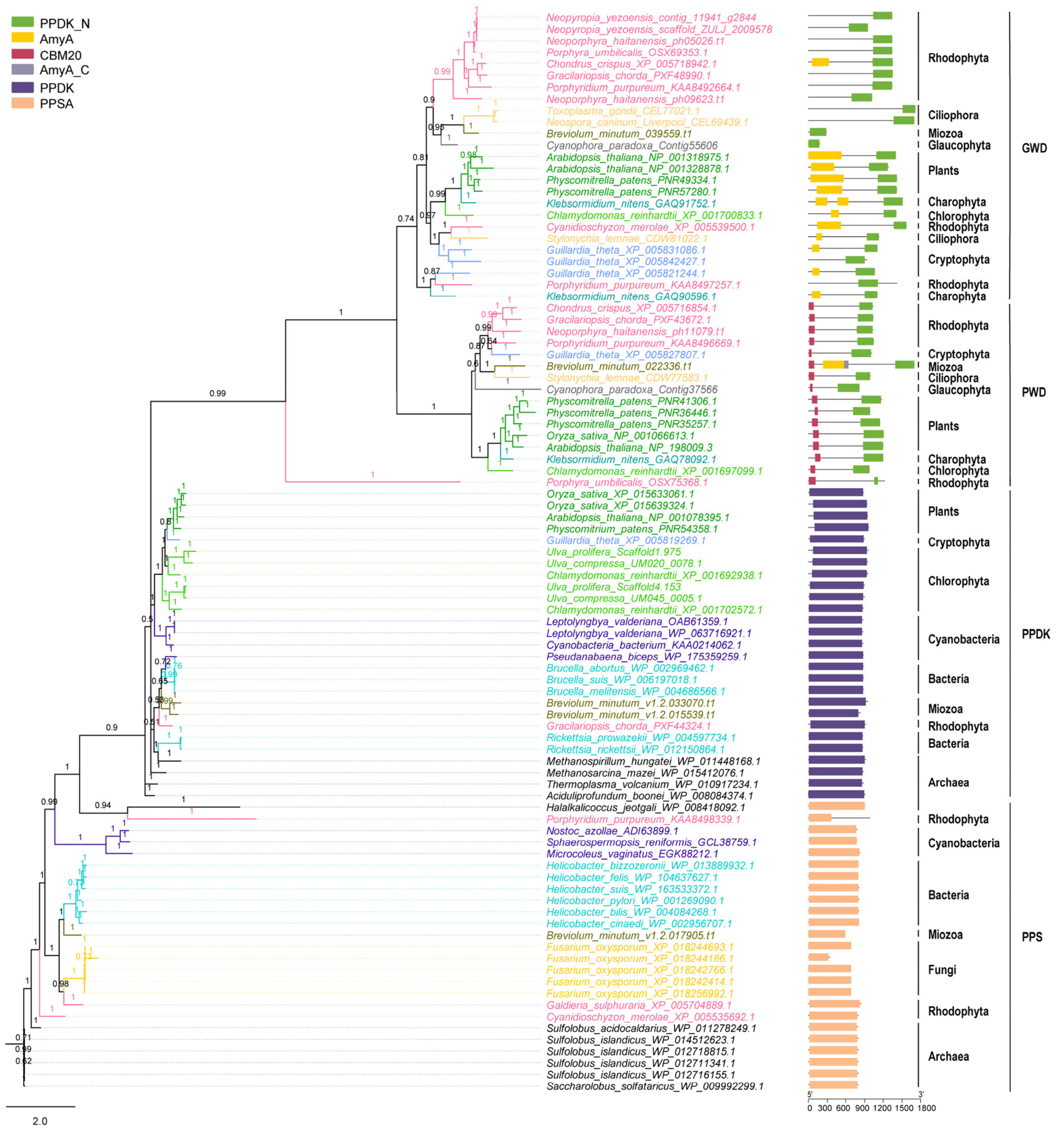

Figure S10. Bayesian phylogenetic tree and domain arrangement of glucan water dikinase (GWD), phosphoglucan water dikinase (PWD), pyruvate phosphate dikinase (PPDK), and pyruvate water dikinase (PPS) based on the amino acid sequences of GWD, PWD, PPDK, and PPS. The GWDs and PWD of *Neoporphyra haitanensis* may have originated from primary endosymbiotic eukaryotic hosts. All protein accession numbers are listed in Table S3. Domain architecture of proteins is shown, and different colored blocks represent different types of conserved domains. Amino acid length scale is shown at the bottom. PPDK_N, pyruvate phosphate dikinase, PEP/pyruvate binding domain; AmyA, alpha-amylase; AmyA_C, alpha-amylase C-terminal beta-sheet domain; CBM20, carbohydrate-binding module 20.

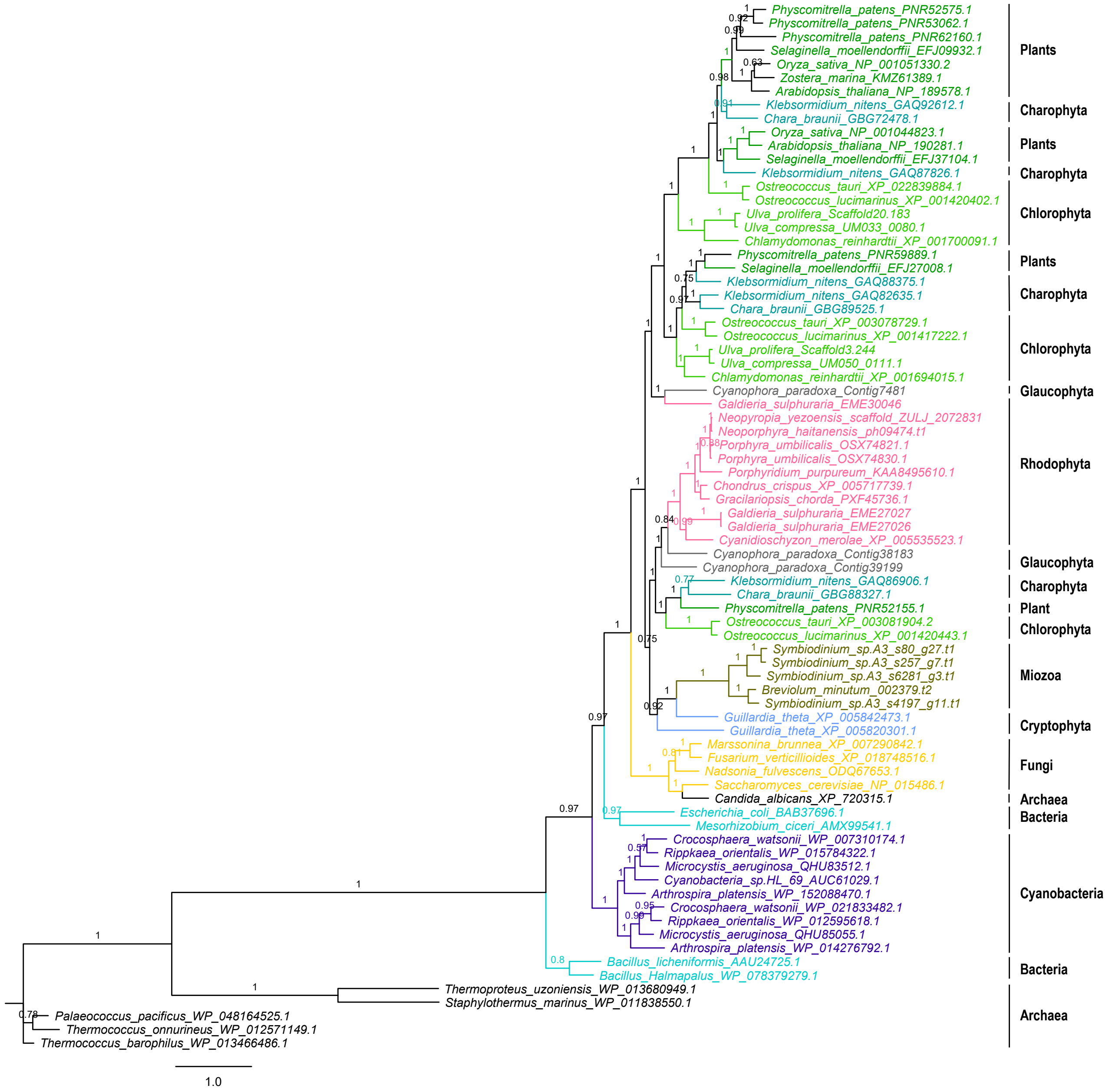

Figure S11. Bayesian phylogenetic tree of phosphorylase (PHO) based on PHO amino acid sequences. The PHO of *Neoporphyra haitanensis* originated from primary endosymbiotic eukaryotic hosts. All protein accession numbers are listed in Table S3.

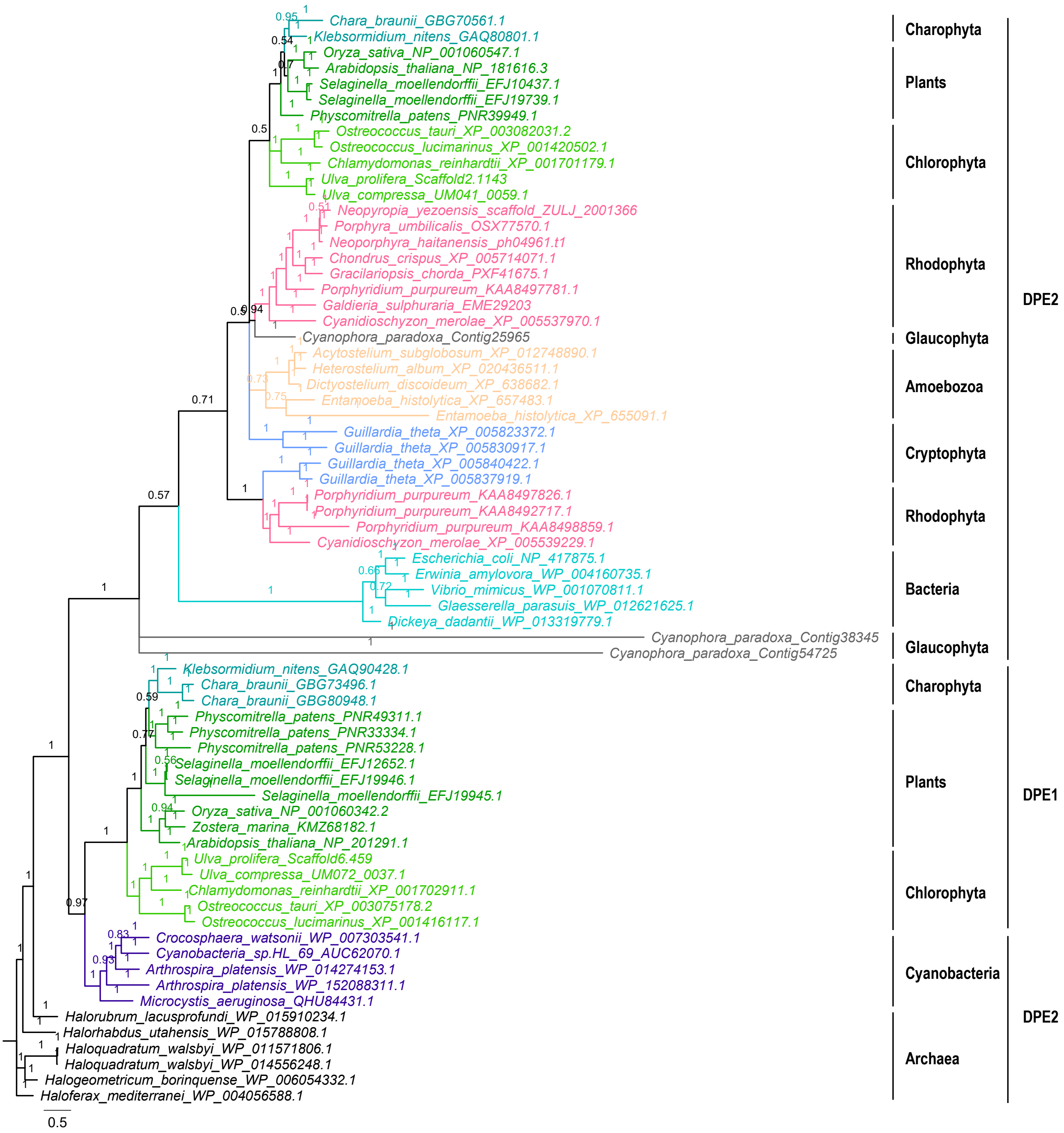

Figure S12. Bayesian phylogenetic tree of disproportionating enzyme (DPE1) and 4-alpha-glucanotransferase (DPE2) based on DPE1 and DPE2 amino acid sequences. The DPE2 of *Neoporphyra haitanensis* may have originated from primary endosymbiotic eukaryotic hosts. All protein accession numbers are listed in Table S3.

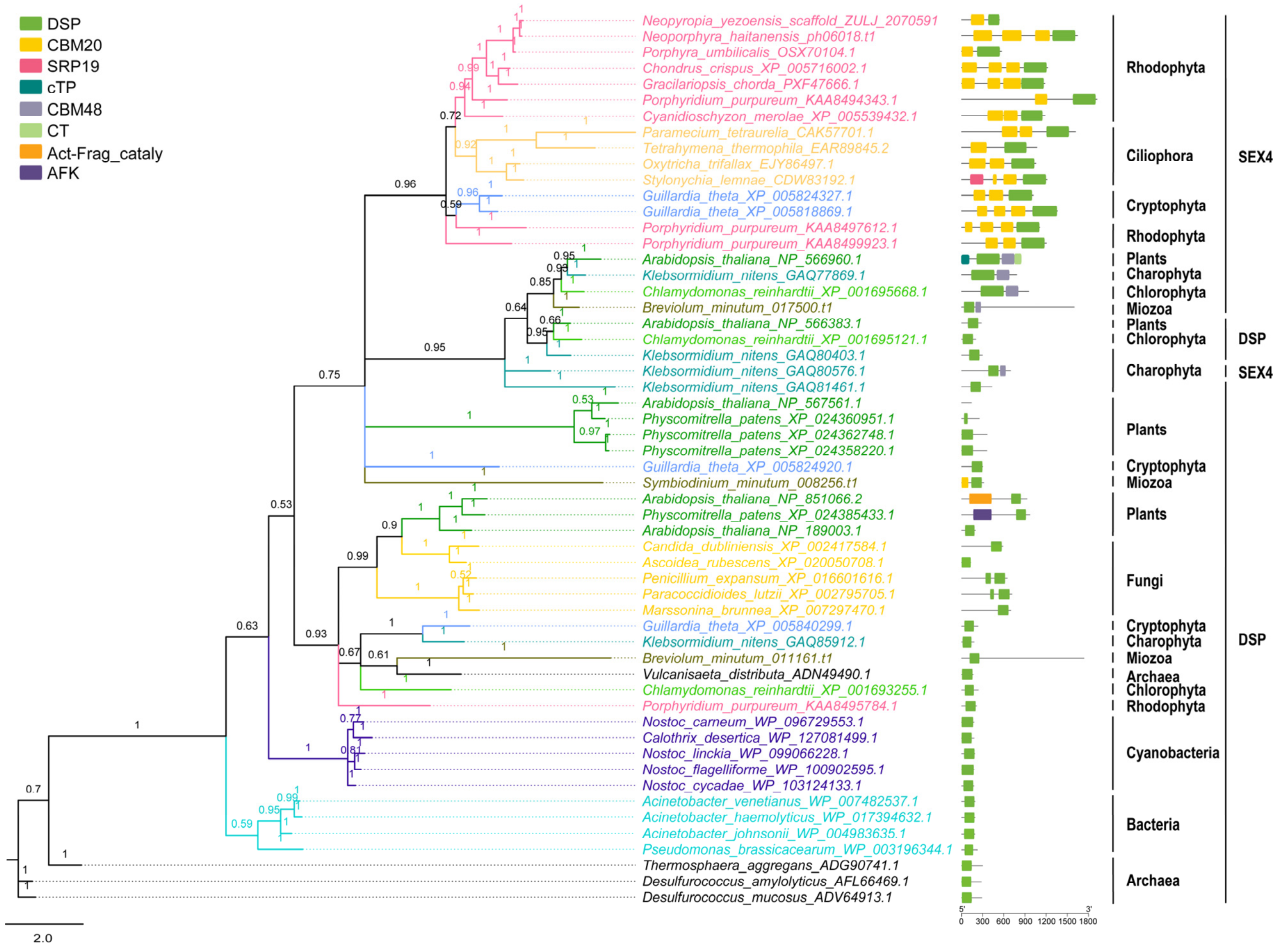

Figure S13. Bayesian phylogenetic tree and domain arrangement of dual-specificity phosphatase starch excess 4 (SEX4) and dual specificity phosphatase (DSP) based on amino acid sequences of SEX4 and DSP amino acid sequences. The SEX4 of *Neoporphyra haitanensis* may have originated from primary endosymbiotic eukaryotic hosts. All sequence information is shown in Supplementary Table 1. Domain architecture of proteins is shown, and different colored blocks represent different types of conserved domains. Amino acid length scale is shown at the bottom. DSP, dual specificity phosphatase domain; CBM20, carbohydrate-binding module 20; SRP19, signal recognition particle 19 protein; cTP, chloroplast transit peptide; CBM48, carbohydrate-binding module 48; CT, C-terminal domain; Act-Frag_cataly, actin-fragmin kinase, catalytic; AFK, actin-fragmin kinase.

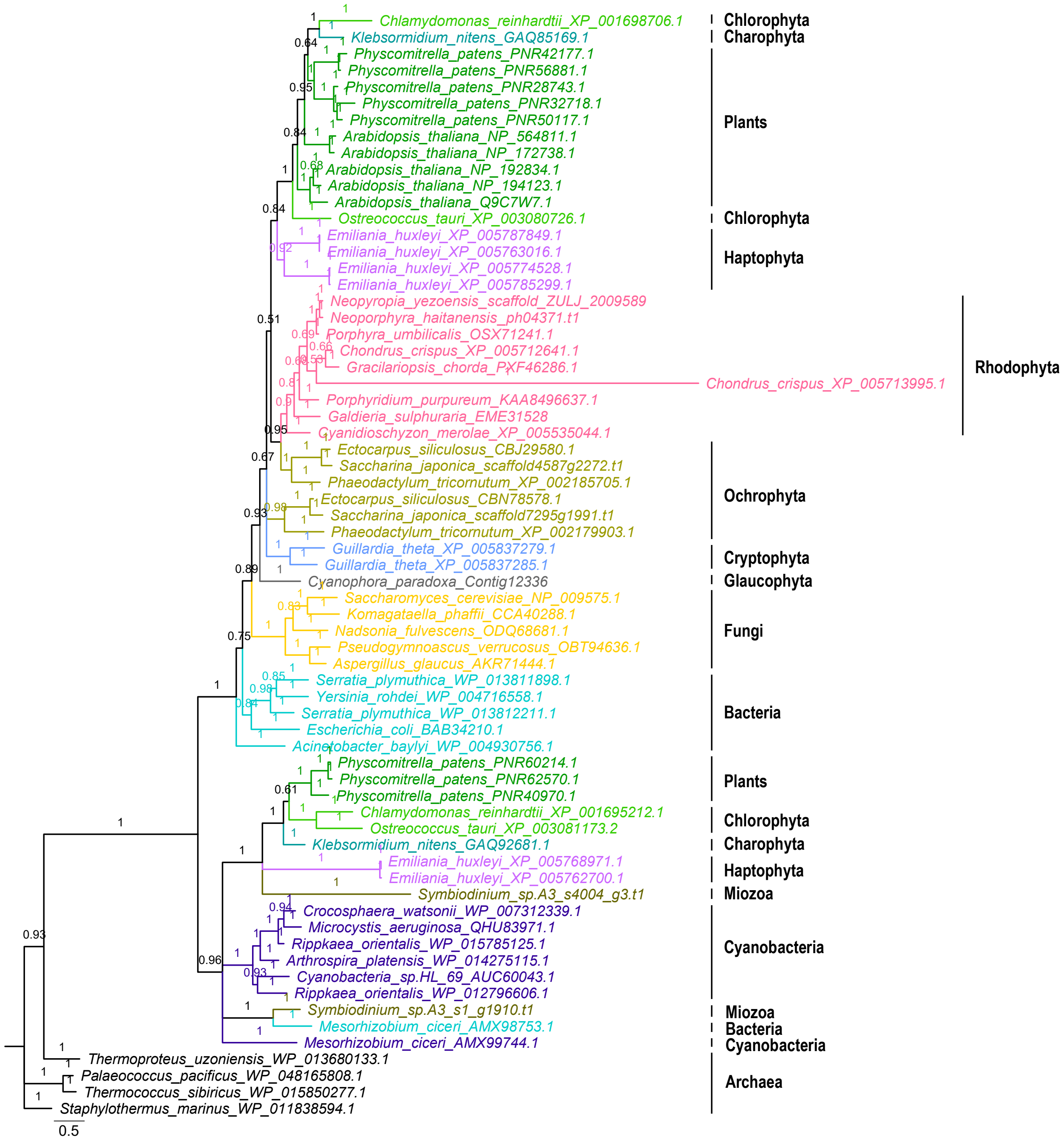

Figure S14. Bayesian phylogenetic tree of UDP-glucose-4-epimerase (GALE) based on GALE amino acid sequences. The GALE of *Neoporphyra haitanensis* originated from primary endosymbiotic eukaryotic hosts. All protein accession numbers are listed in Table S3.

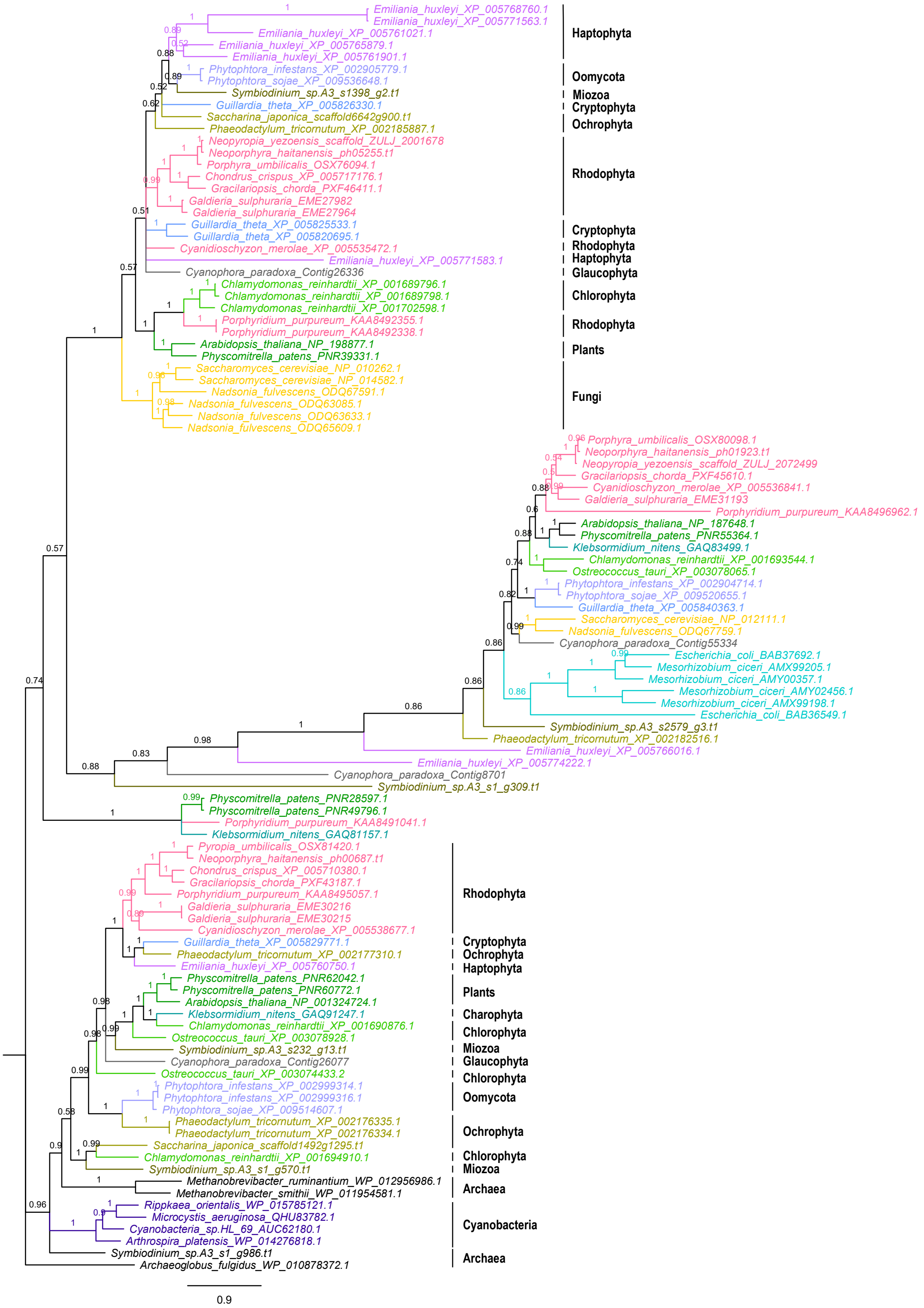

Figure S15. Bayesian phylogenetic tree of glycerol-3-phosphate dehydrogenase (GPDH) based on GPDH amino acid sequences. The GPDHs of *Neoporphyra haitanensis* seemed that there were two origins, one was primary endosymbiotic eukaryotic hosts and the other cannot be confirmed. All protein accession numbers are listed in Table S3.

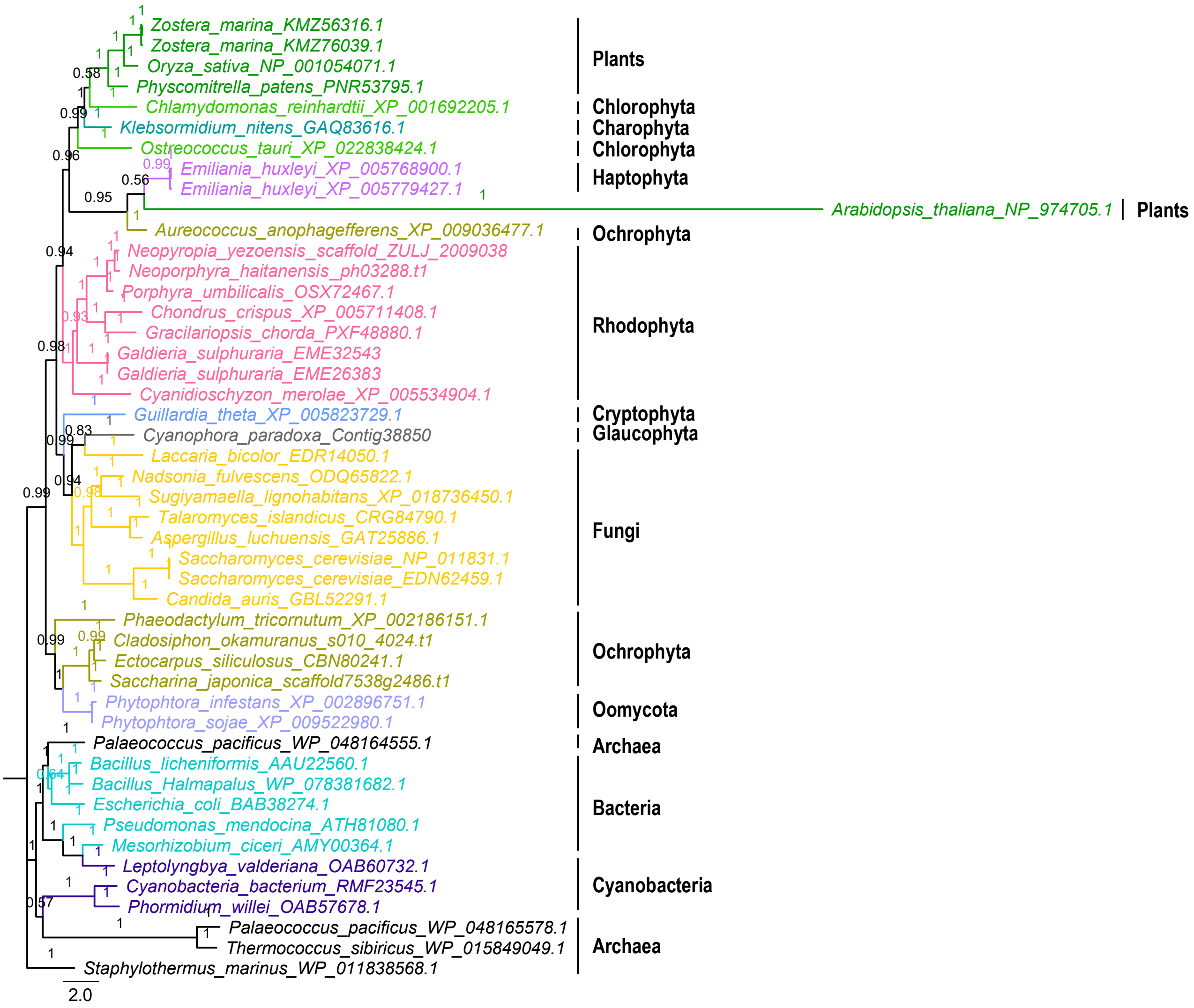

Figure S16. Bayesian phylogenetic tree of glycerol kinase (GK) based on GK amino acid sequences. The GK of *Neoporphyra haitanensis* originated from primary endosymbiotic eukaryotic hosts. All protein accession numbers are listed in Table S3.

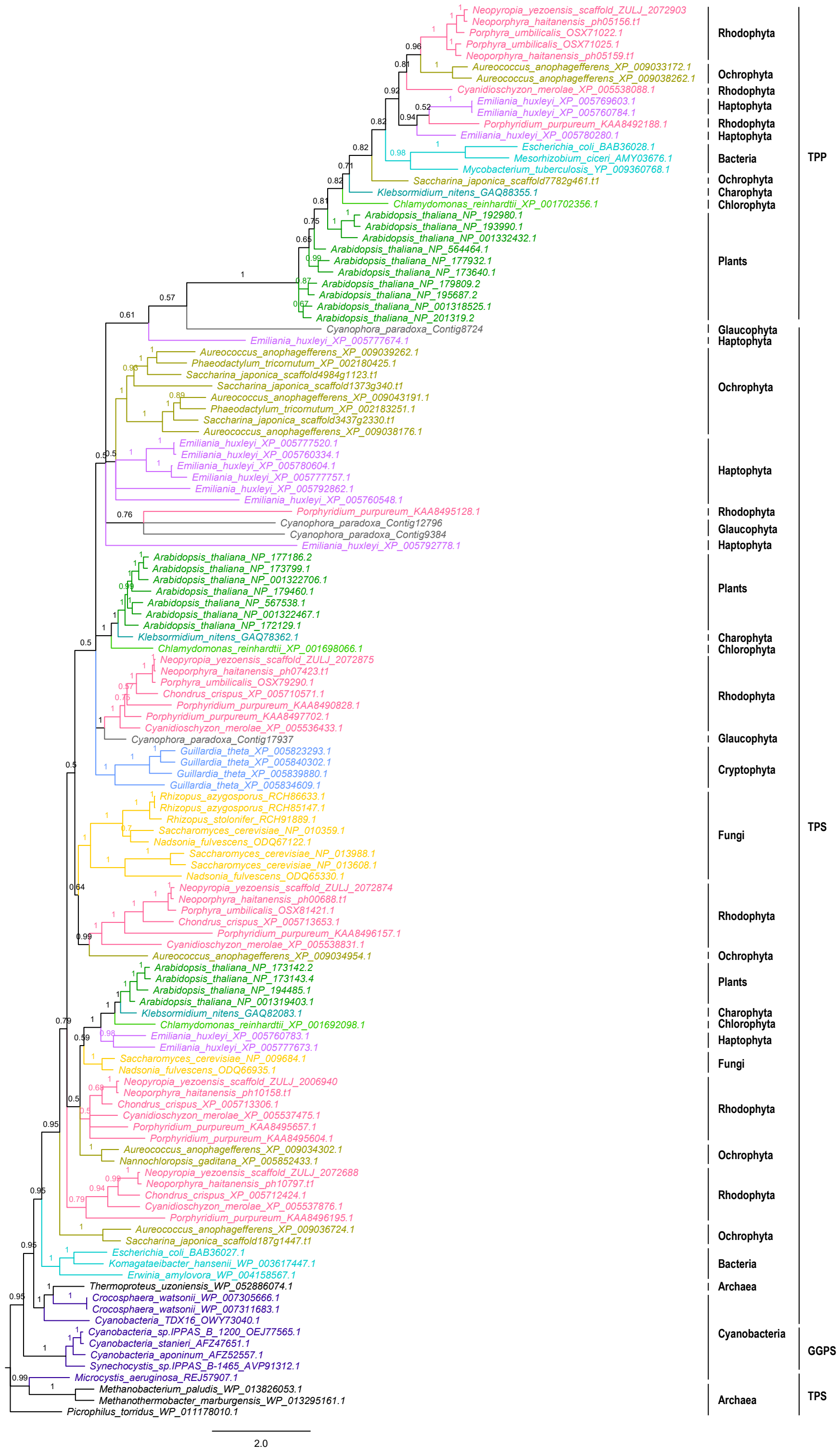

Figure S17. Bayesian phylogenetic tree of trehalose-6-phosphate synthase (TPS), trehalose-6-phosphate phosphatase (TPP), and glucosyl-glycerol phosphate synthase (GGPS) based on TPS, TPP, and GGPS amino acid sequences. The TPSs of *Neoporphyra haitanensis* may have originated from primary endosymbiotic eukaryotic hosts and TPPs of *Neoporphyra haitanensis* may have originated from bacteria via horizontal gene transfer. All protein accession numbers are listed in Table S3.

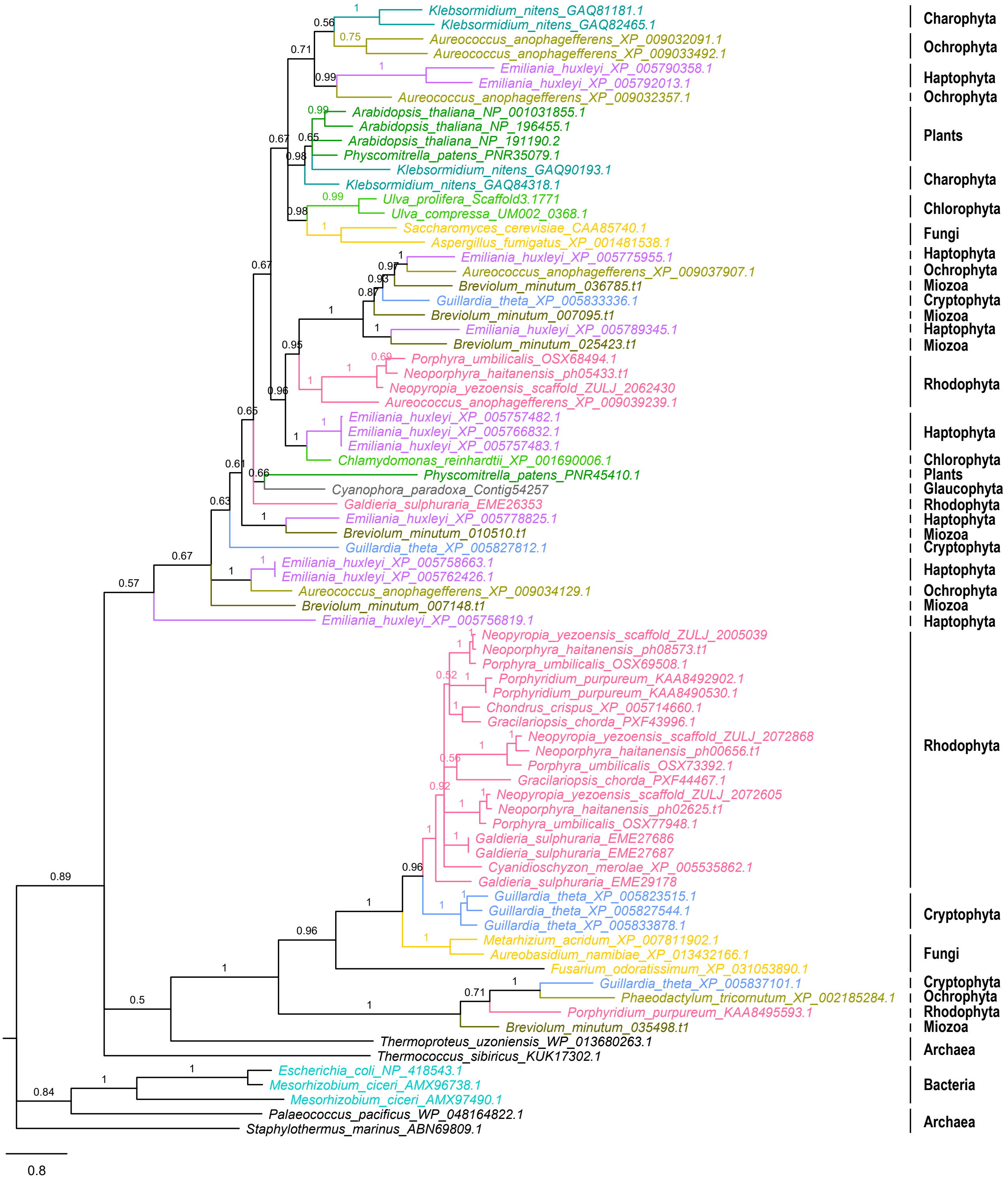

Figure S18. Bayesian phylogenetic tree of alpha-galactosidase (GLA) based on GLA amino acid sequences. The GLAs of *Neoporphyra haitanensis* may have originated from primary endosymbiotic eukaryotic hosts. All protein accession numbers are listed in Table S3.

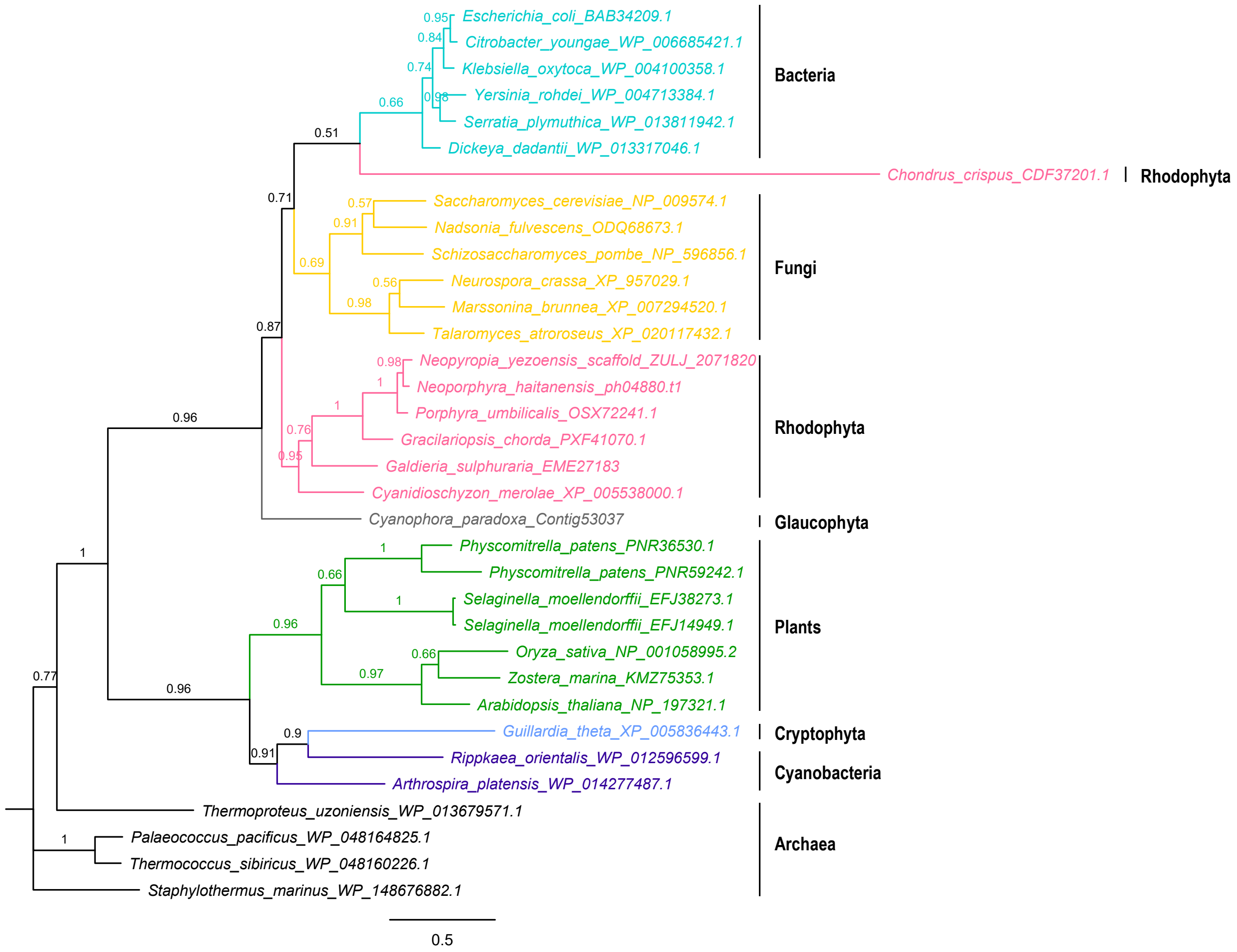

Figure S19. Bayesian phylogenetic tree of galactose-1-phosphate uridylyltransferase (GALT) based on GALT amino acid sequences. The GALT of *Neoporphyra haitanensis* originated from primary endosymbiotic eukaryotic hosts. All protein accession numbers are listed in Table S3.

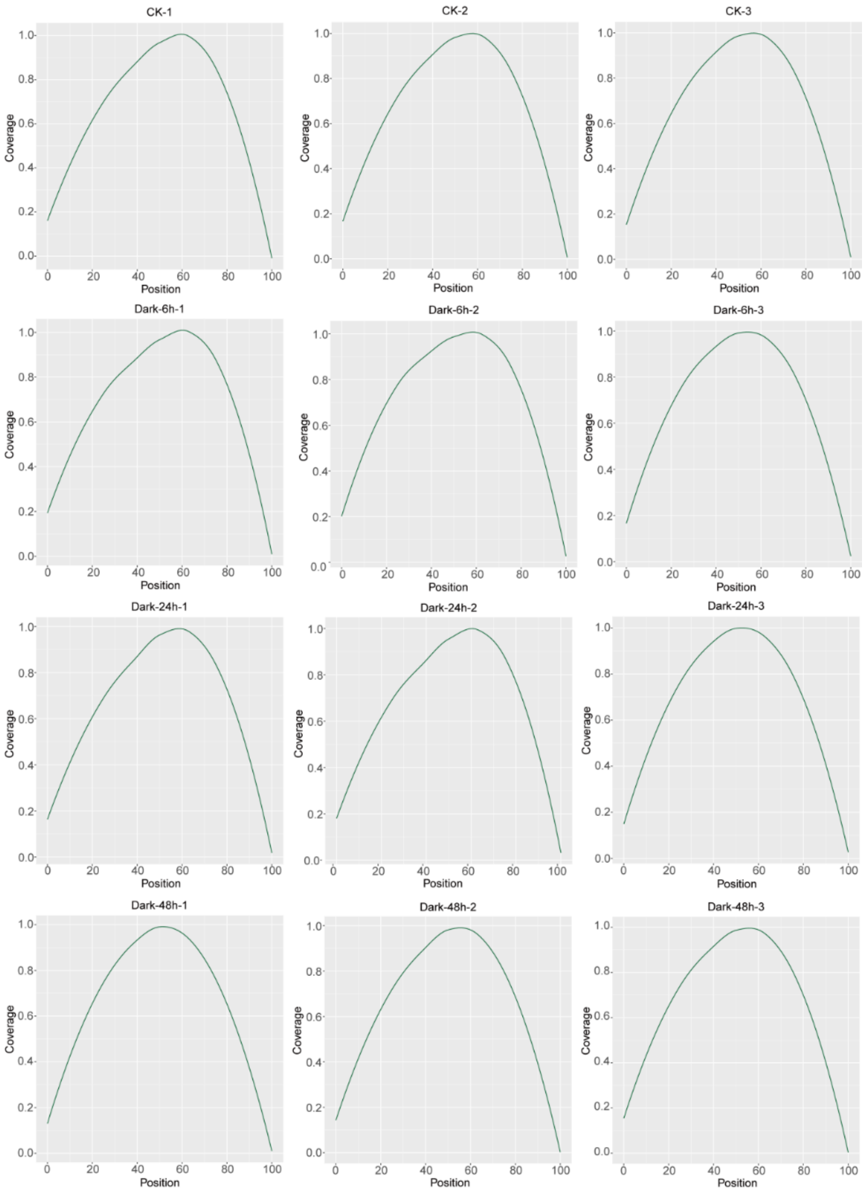

Figure S20. Diagram of gene coverage analysis results. The abscissa shows the percentage of individual gene base length in the total base length, whereas 0 and 100 represent the 5' and 3' ends of the gene, respectively. The ordinate shows the sum of the number of sequences aligned to the corresponding interval on the horizontal axis of all genes.

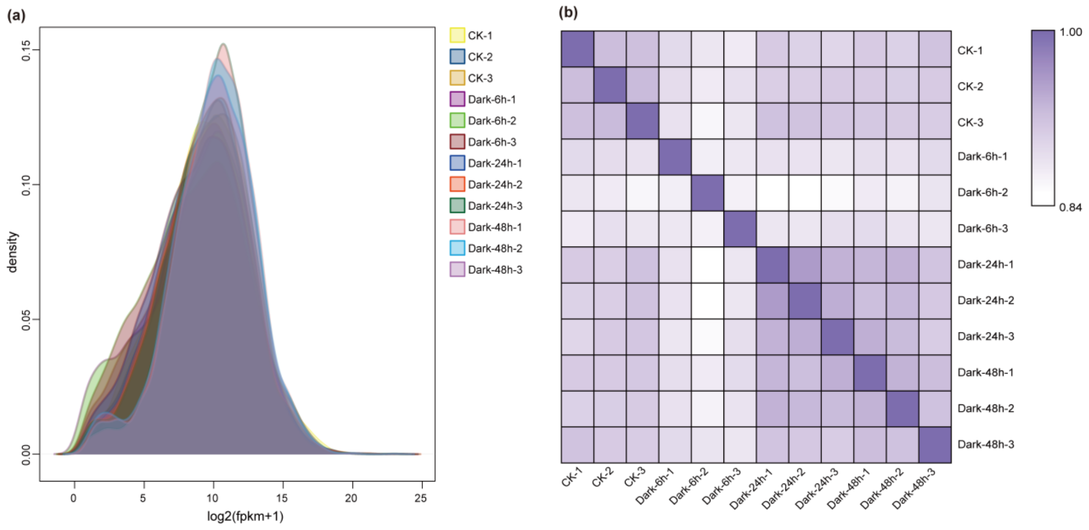

Figure S21. Sequencing quality detection. (a) Fragments per kilobase of exon per million mapped reads (FPKM) distribution in all samples. Different-colored curves represent different samples, the abscissa of the points on the curve represents the logarithm of the corresponding sample FPKM plus 1, and the ordinate of the points represents the probability density. (b) Pearson correlation between samples. The correlation coefficients between the samples are indicated by different color shades. CK, control.

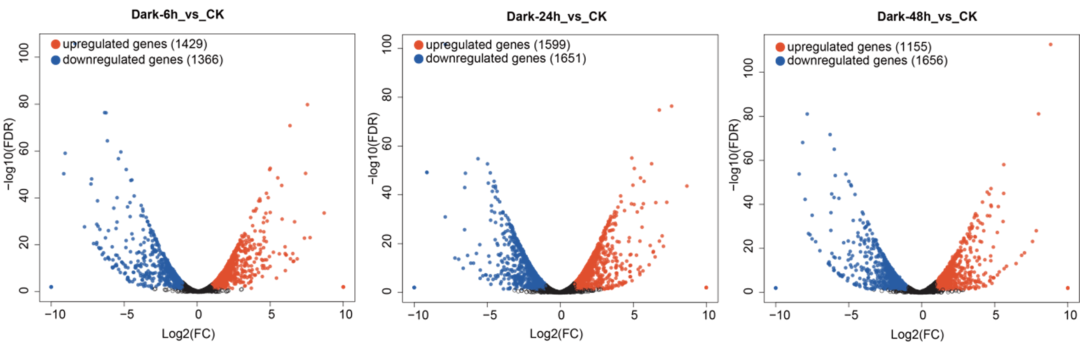

Figure S22. Volcano plots of differentially expressed genes (DEGs). The x-axis indicates the fold change of gene expression between different samples, and the y-axis indicates the statistical significance of the differences. Red, blue, and black points represent significantly upregulated, downregulated, and non-regulated genes, respectively. CK, control.

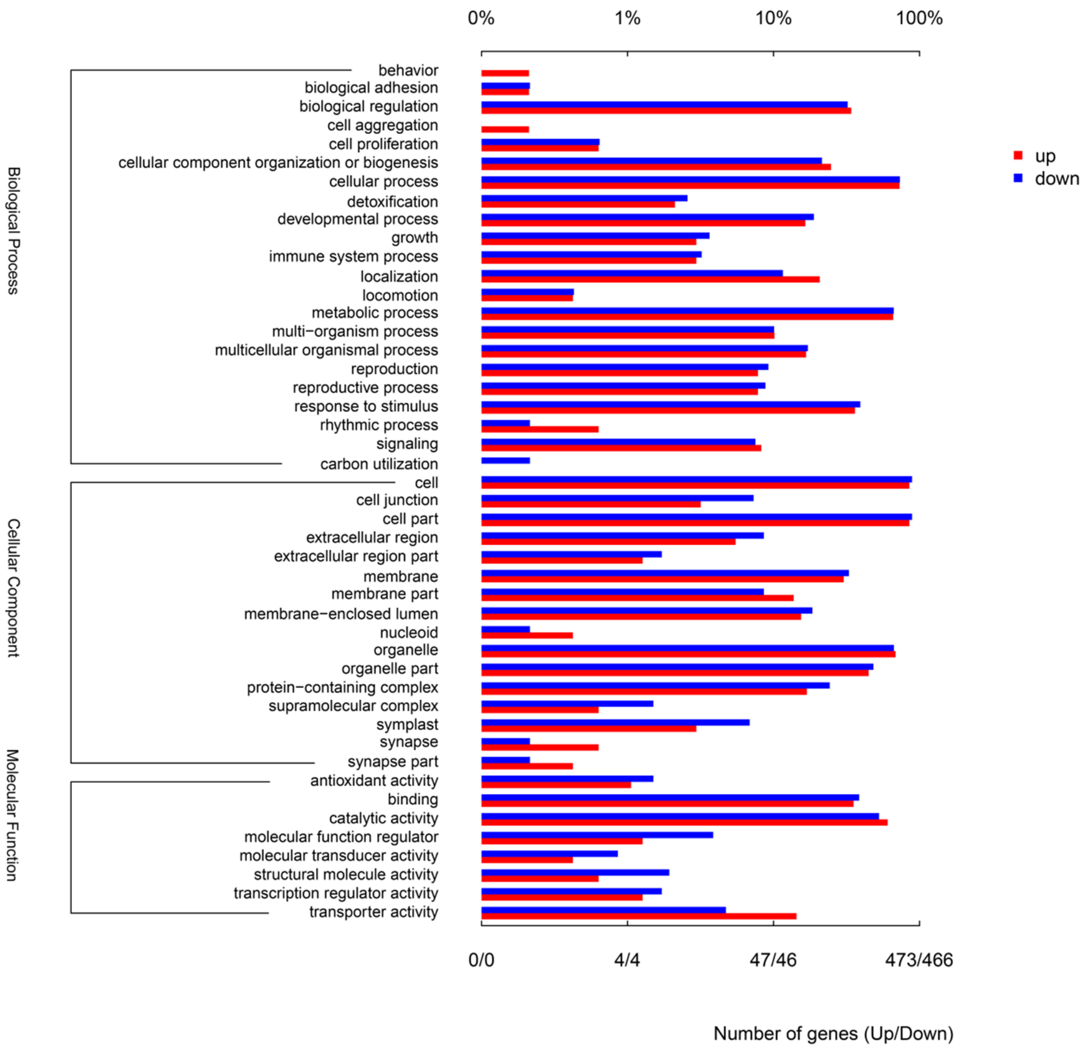

Figure S23. Gene ontology (GO) functional annotation of differentially expressed genes (DEGs) of *Neoporphyra haitanensis* transcriptome under 6 h of dark vs under control (CK). Red bars represent upregulated genes and blue bars represent downregulated genes.

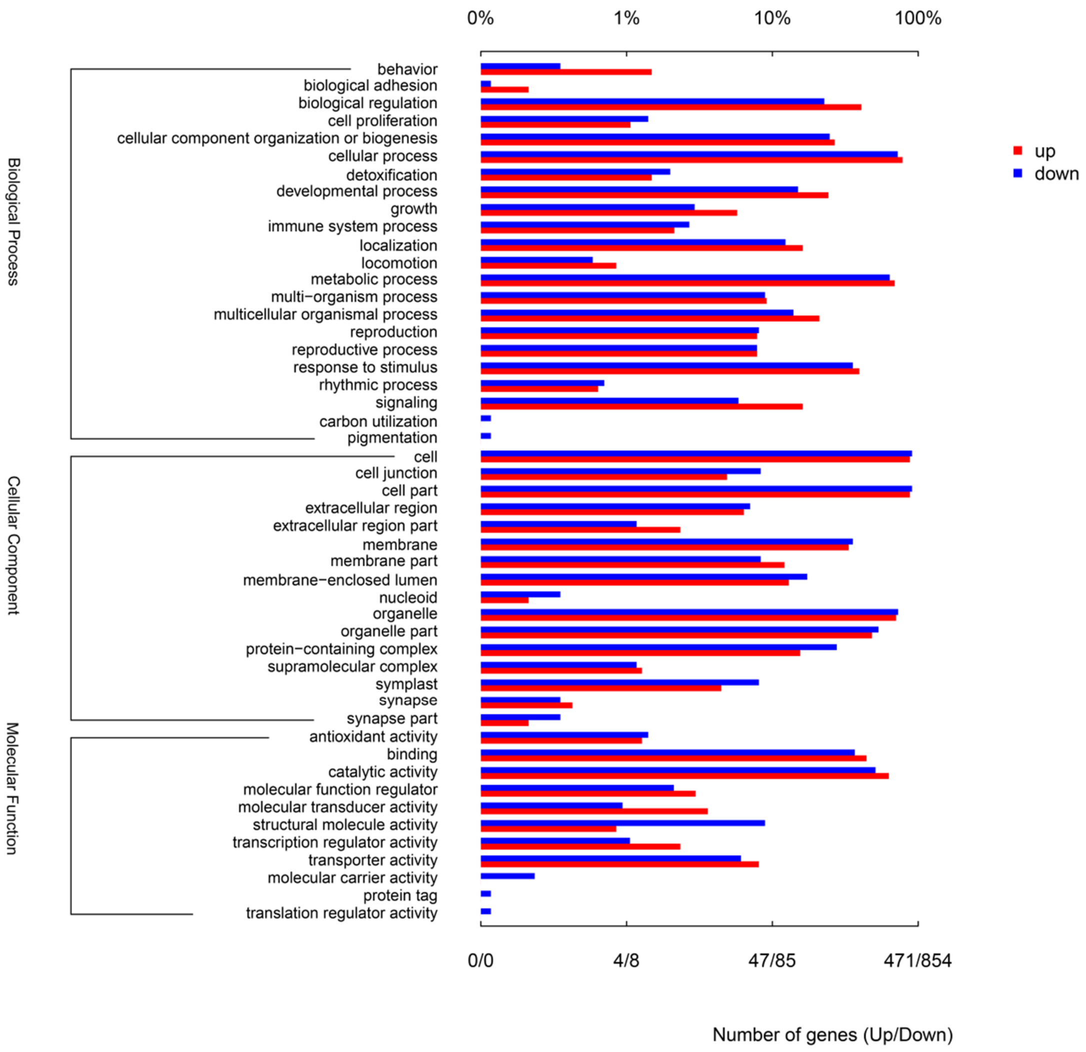

Figure S24. Gene ontology (GO) functional annotation of differentially expressed genes (DEGs) of *Neoporphyra haitanensis* transcriptome under 24 h of dark vs under control (CK). Red bars represent upregulated genes and blue bars represent downregulated genes.

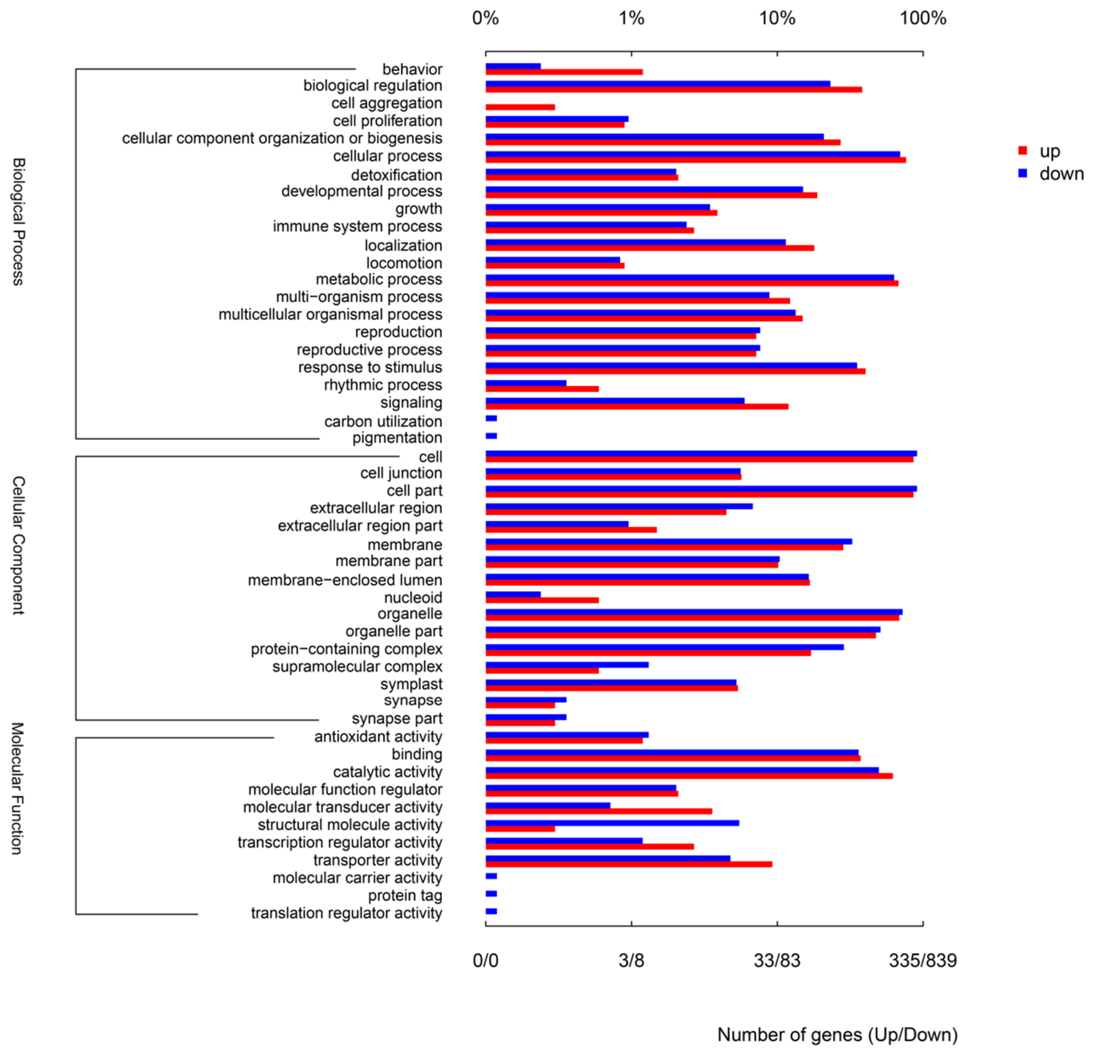

Figure S25. Gene ontology (GO) functional annotation of differentially expressed genes (DEGs) of *Neoporphyra haitanensis* transcriptome under 48 h of dark vs under control (CK). Red bars represent upregulated genes and blue bars represent downregulated genes.

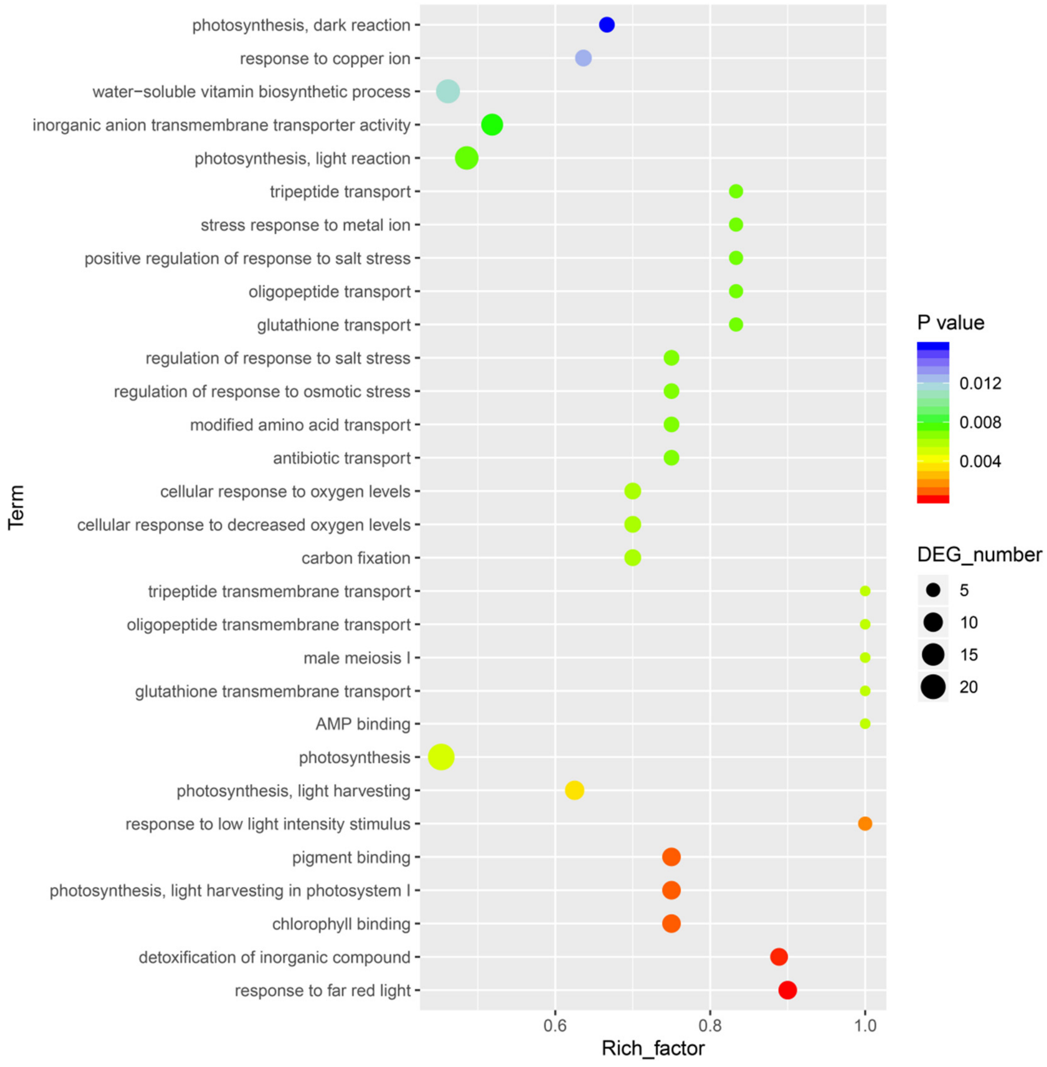

Figure S26. Gene ontology (GO) functional enrichment of differentially expressed genes (DEGs) of *Neoporphyra haitanensis* transcriptome under 6 h of dark vs under control (CK). The horizontal axis represents the enrichment factor, the ordinate represents the GO term, the point size indicates the DEG number, and the color atlas from blue to red represents uncorrected P-values.

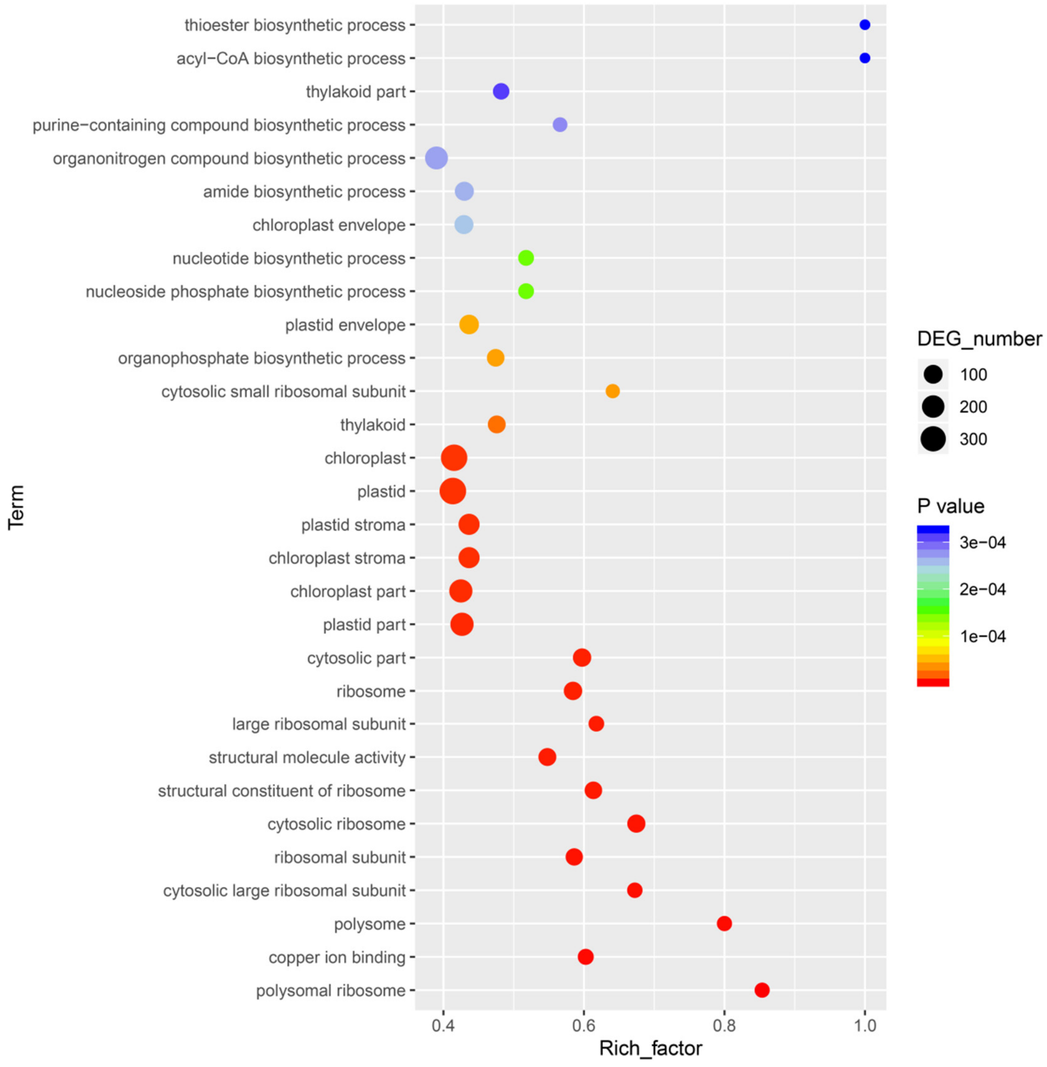

Figure S27. Gene ontology (GO) functional enrichment of differentially expressed genes (DEGs) of *Neoporphyra haitanensis* transcriptome under 24 h of dark vs under control (CK). The horizontal axis represents the enrichment factor, the ordinate represents the GO term, the point size indicates the DEG number, and the color atlas from blue to red represents uncorrected P-values.

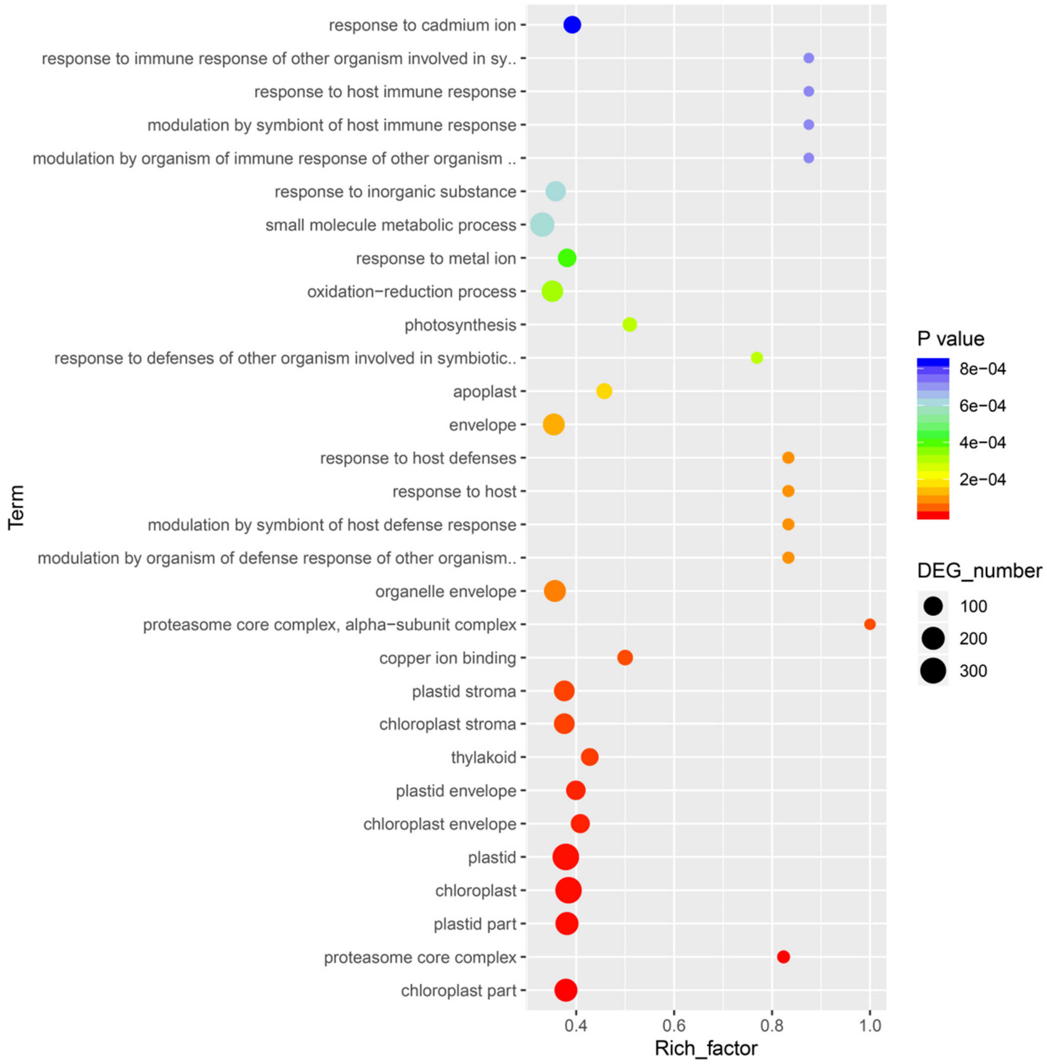

Figure S28. Gene ontology (GO) functional enrichment of differentially expressed genes (DEGs) of *Neoporphyra haitanensis* transcriptome under 48 h of dark vs under control (CK). The horizontal axis represents the enrichment factor, the ordinate represents the GO term, the point size indicates the DEG number, and the color atlas from blue to red represents uncorrected P-values.

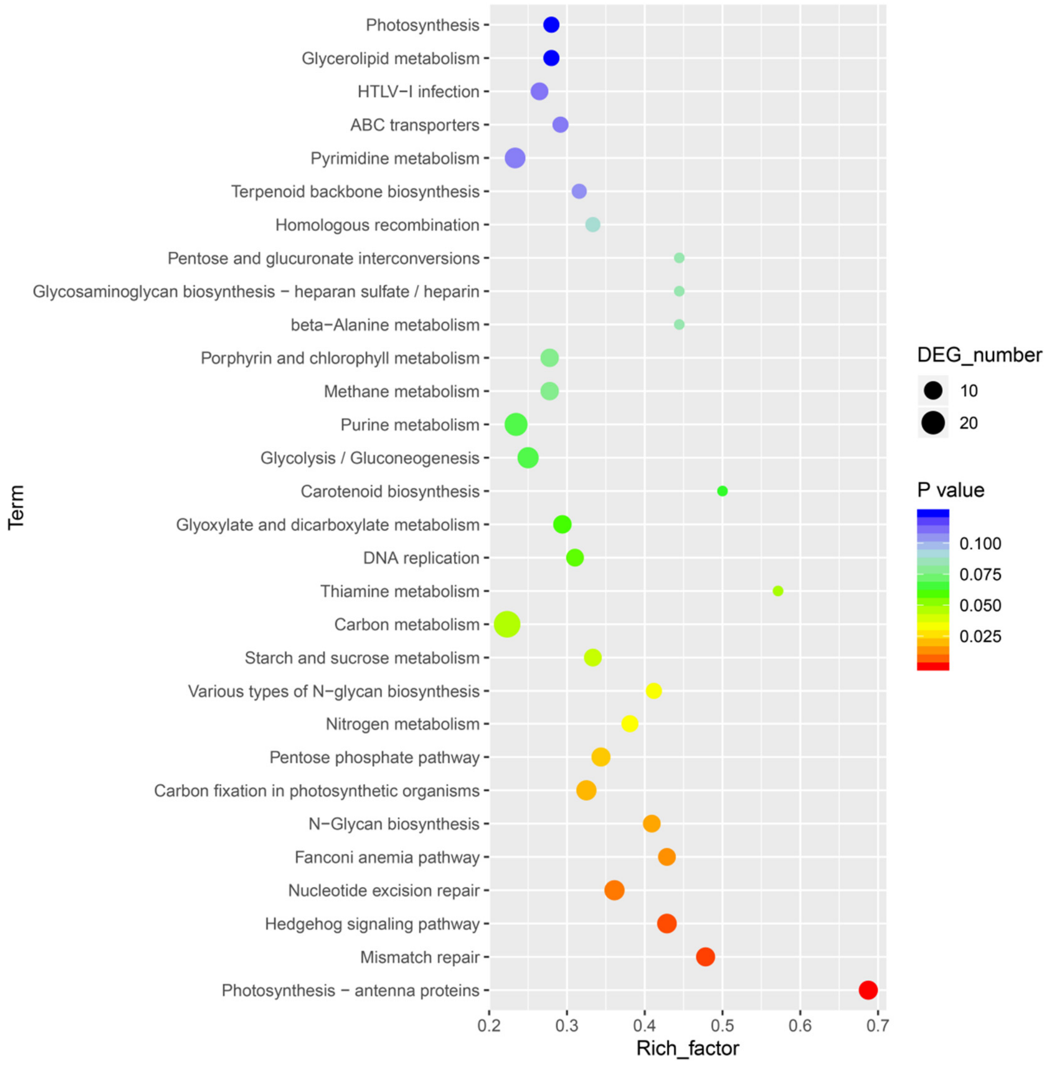

Figure S29. Pathway functional enrichment analysis of differentially expressed genes (DEGs) of *Neoporphyra haitanensis* transcriptome under 6 h of dark vs under control (CK). The horizontal axis represents the enrichment factor, the ordinate represents the pathway name, the point size indicates the DEG number, and the color atlas from blue to red represents uncorrected P-values.

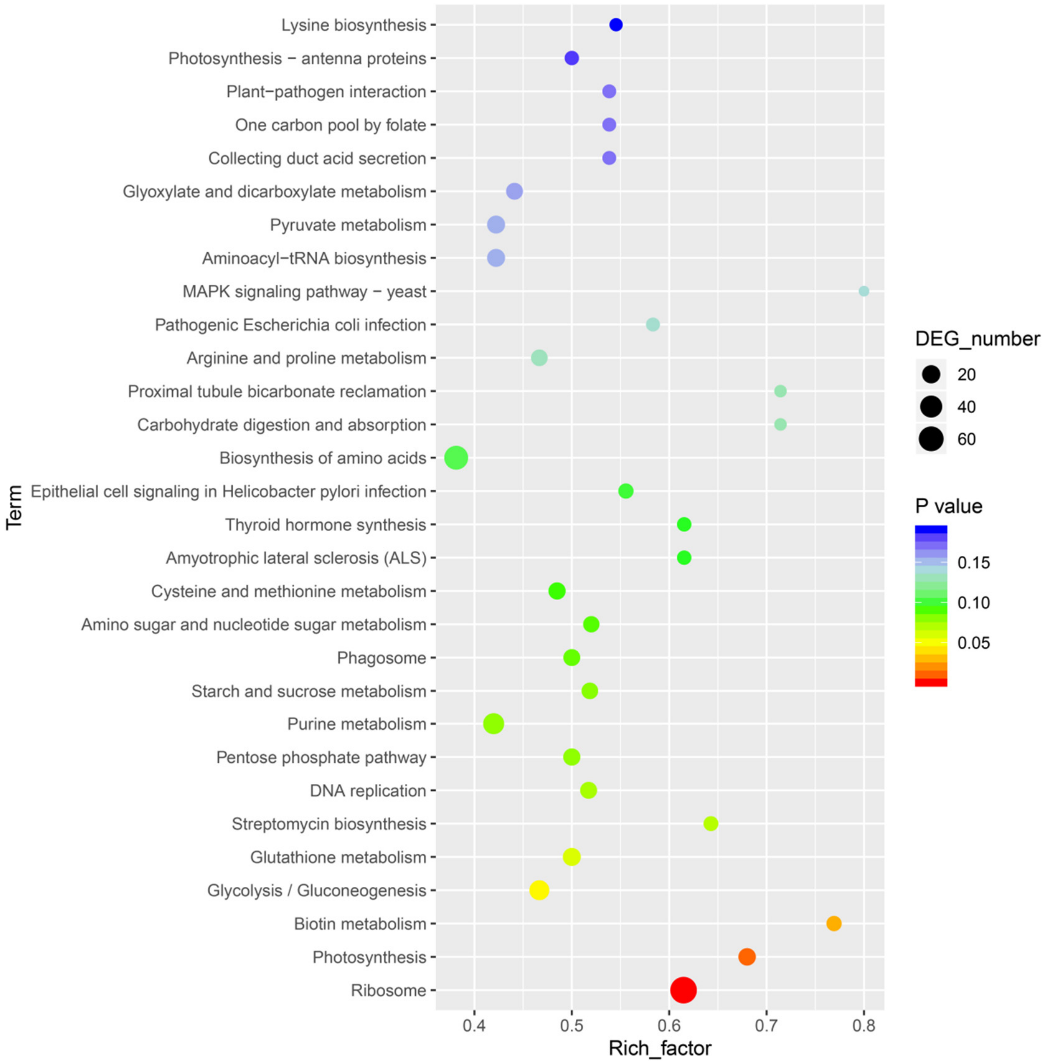

Figure S30. Pathway functional enrichment analysis of differentially expressed genes (DEGs) of *Neoporphyra haitanensis* transcriptome under 24 h of dark vs under control (CK). The horizontal axis represents the enrichment factor, the ordinate represents the pathway name, the point size indicates the DEG number, and the color atlas from blue to red represents uncorrected P-values.

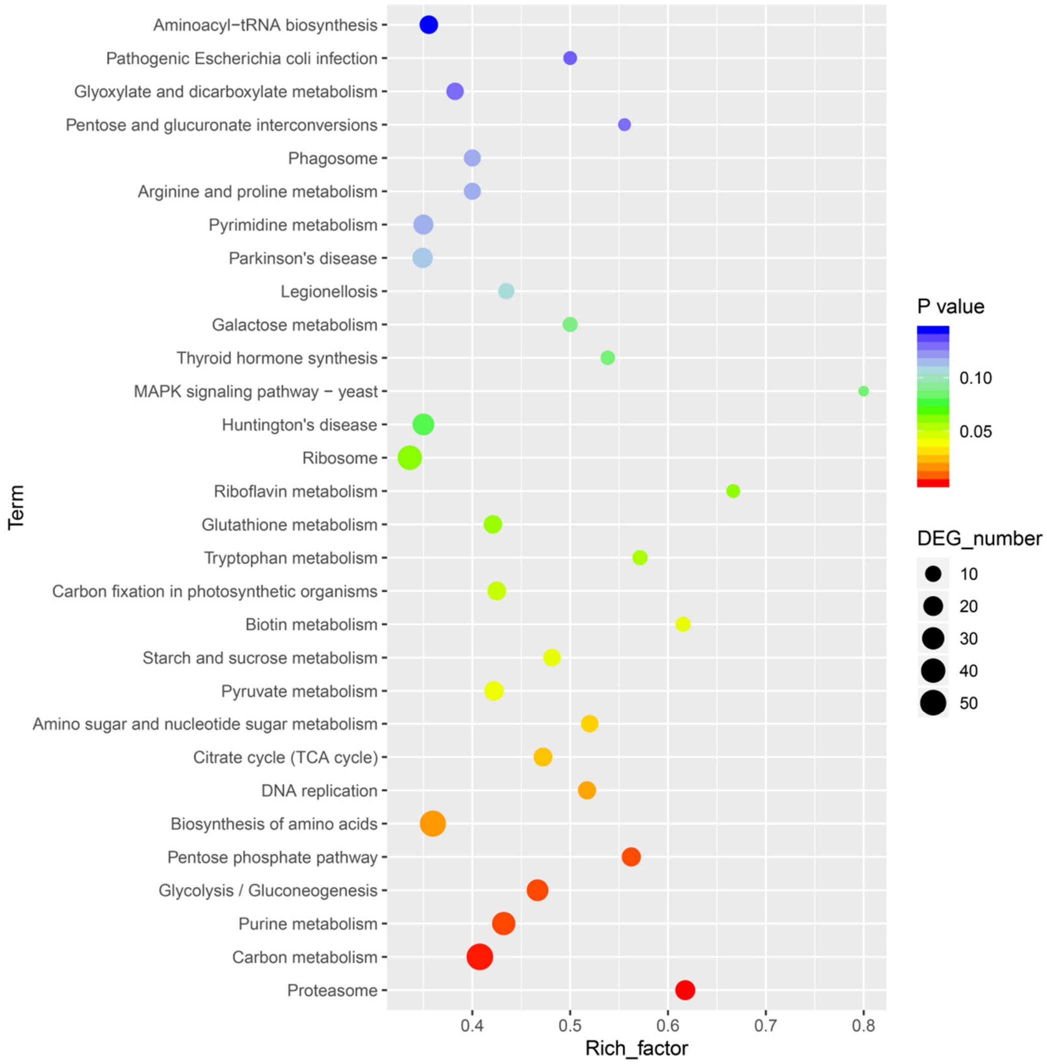

Figure S31. Pathway functional enrichment analysis of differentially expressed genes (DEGs) of *Neoporphyra haitanensis* transcriptome under 48 h of dark vs under control (CK). The horizontal axis represents the enrichment factor, the ordinate represents the pathway name, the point size indicates the DEG number, and the color atlas from blue to red represents uncorrected P-values.

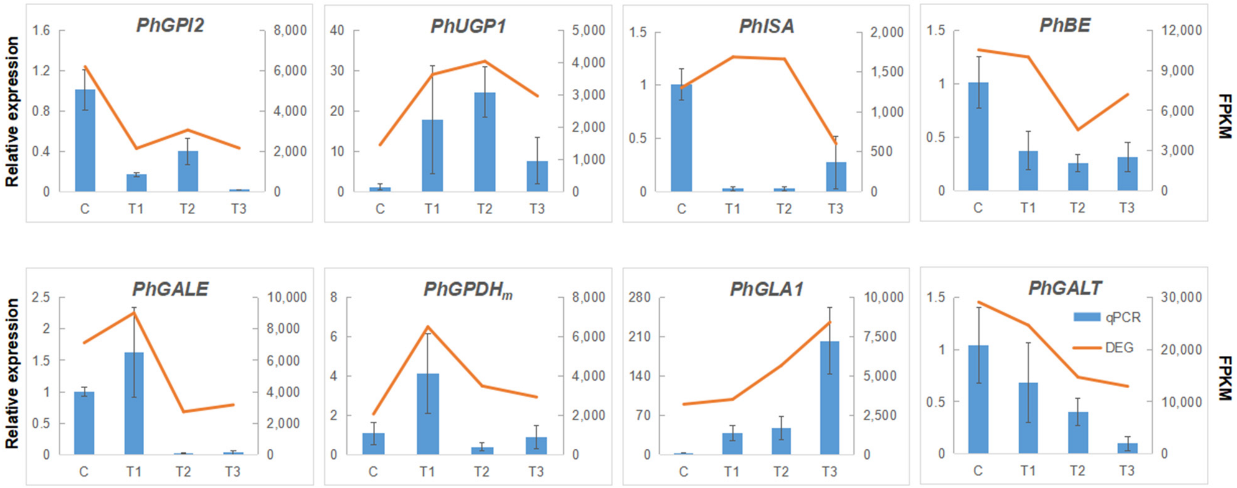

Figure S32. qRT-PCR validation of RNA-seq at 8 selected target genes. Abbreviations: C, control; T1, 6 h after darkness; T2, 24 h after darkness; T3, 48 h after darkness; qPCR, qRT-PCR; FPKM, the fragments per kilobase of exon per million mapped reads.

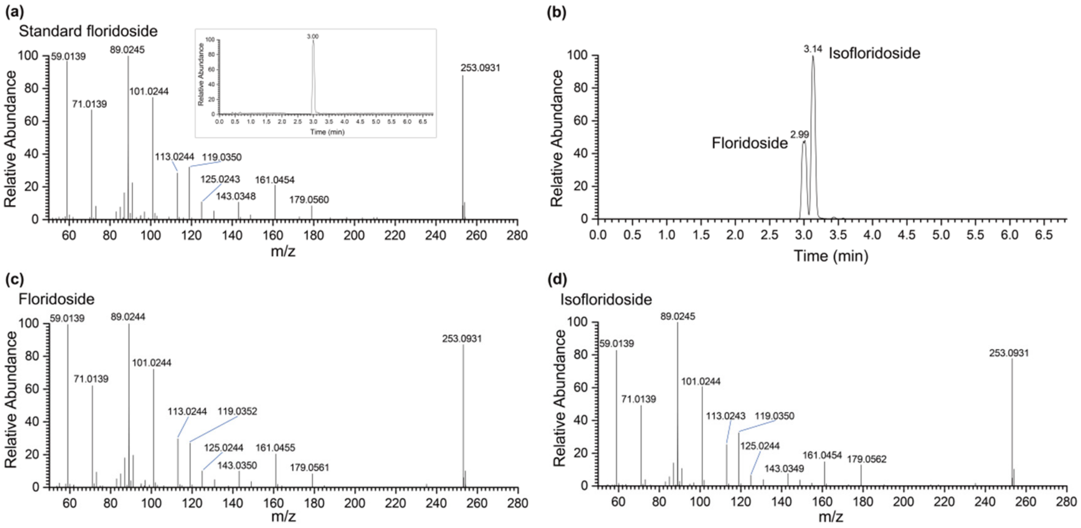

Figure S33. LC-MS analysis of floridoside and isofloridoside. (a) The chromatogram map and mass spectrum of floridoside standard (peak at 2.99 min, [M]-=253.0935). (b) Representative liquid chromatography separation of purified floridoside (peak at 2.99 min) and isofloridoside samples (peak at 3.14 min). (c) Mass spectrum of floridoside peak (2.99 min). (d) Mass spectrum of isofloridoside peak (3.14 min).

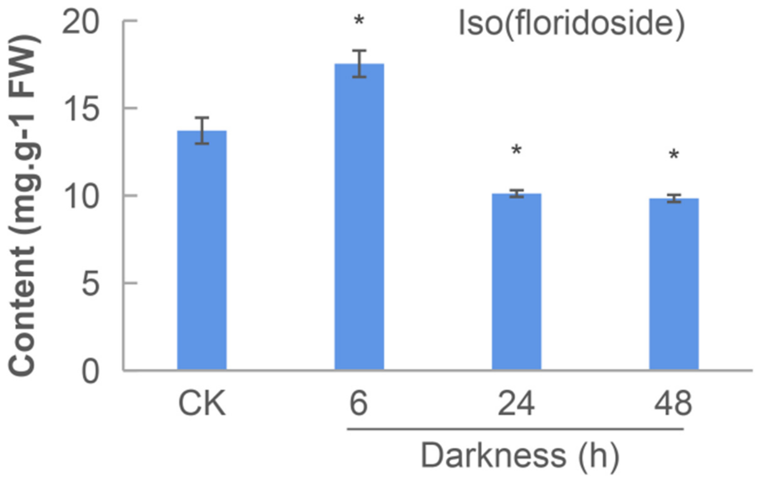

Figure S34. Total floridoside and isofloridoside contents (three replicates per treatment). Error bars indicate the standard deviation (SD) for three measurements. Asterisks indicate significant differences between darkness and the control for each group ($P < 0.05$). CK, control; FW, fresh weight.
